# *In Vivo* Photoacoustic Monitoring of Stem Cell Location and Apoptosis with Caspase-3-Responsive Nanosensors

**DOI:** 10.1021/acsnano.3c04161

**Published:** 2023-09-13

**Authors:** Anamik Jhunjhunwala, Jinhwan Kim, Kelsey P. Kubelick, C. Ross Ethier, Stanislav Y. Emelianov

**Affiliations:** † Wallace H. Coulter Department of Biomedical Engineering, Georgia Institute of Technology and Emory University School of Medicine, Atlanta, Georgia 30332, United States; ‡ School of Electrical & Computer Engineering, Georgia Institute of Technology, Atlanta, Georgia 30332, United States

**Keywords:** nanosensors, cell engineering, stem cells, cell apoptosis, photoacoustic imaging, cell tracking

## Abstract

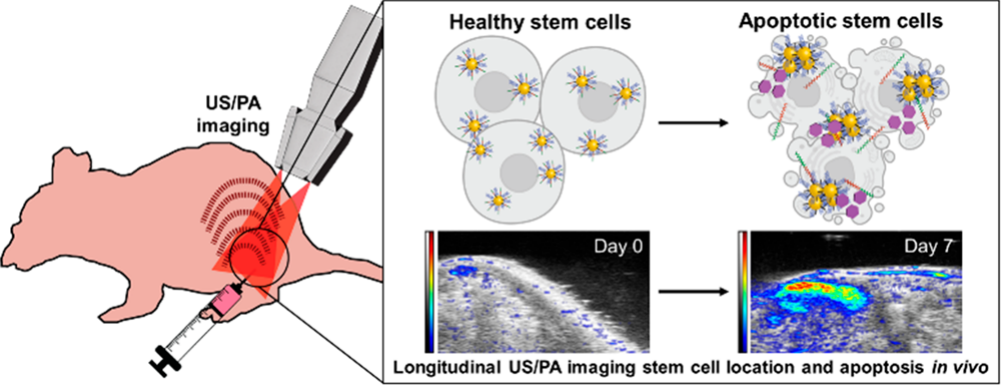

Stem cell therapy has immense potential in a variety of regenerative medicine applications. However, clinical stem cell therapy is severely limited by challenges in assessing the location and functional status of implanted cells *in vivo*. Thus, there is a great need for longitudinal, noninvasive stem cell monitoring. Here we introduce a multidisciplinary approach combining nanosensor-augmented stem cell labeling with ultrasound guided photoacoustic (US/PA) imaging for the spatial tracking and functional assessment of transplanted stem cell fate. Specifically, our nanosensor incorporates a peptide sequence that is selectively cleaved by caspase-3, the primary effector enzyme in mammalian cell apoptosis; this cleavage event causes labeled cells to show enhanced optical absorption in the first near-infrared (NIR) window. Optimization of labeling protocols and spectral characterization of the nanosensor *in vitro* showed a 2.4-fold increase in PA signal from labeled cells during apoptosis while simultaneously permitting cell localization. We then successfully tracked the location and apoptotic status of mesenchymal stem cells in a mouse hindlimb ischemia model for 2 weeks *in vivo*, demonstrating a 4.8-fold increase in PA signal and spectral slope changes in the first NIR window under proapoptotic (ischemic) conditions. We conclude that our nanosensor allows longitudinal, noninvasive, and nonionizing monitoring of stem cell location and apoptosis, which is a significant improvement over current end-point monitoring methods such as biopsies and histological staining of excised tissue.

## Introduction

Stem cell therapy has shown great promise for regenerating and/or remodeling damaged or degenerating tissues.^1−4^ Conventional approaches such as transplantation of intact organs require invasive surgery and often fail due to host immunity and incompatibility.^5^ In contrast, autologous stem cell therapy offers a minimally invasive and immune-compatible therapeutic approach and, thus, has been regarded as the next-generation of regenerative medicine.^6,7^ So far, promising preclinical and clinical outcomes of stem cell therapy have been reported for various pathologies, including musculoskeletal and spinal cord injuries, cardiac tissue damage, Parkinson’s disease, and multiple sclerosis.^4,8−11^ However, the translation of these stem cell therapies is severely hindered by issues such as incorrect injection location, poor retention of transplanted cells, poor stem cell viability, and lack of stem cell fate monitoring *in vivo*.^12−16^

The general workflow for autologous stem cell therapy involves harvesting healthy stem cells from a patient, expanding cells in culture, injecting them back into the patient, and evaluating therapeutic outcomes.^17^ However, methods to assess therapeutic outcomes during the course of treatment are lacking, and the therapeutic mechanisms are not easily monitored or well understood.^18−20^ Assessment of outcomes is based on invasive end-point analyses such as histology and biomarker assays, which damage regenerating tissues and do not capture the real-time dynamics of the tissue microenvironment.^14,21^ Thus, we need tools to (1) accurately guide stem cell delivery to target regions and (2) perform spatial and functional tracking of injected cells longitudinally to better understand their biological status *in vivo*. Previously our group reported an image-guided approach to address the first point.^9,22−26^ Other groups have also used a variety of approaches to track stem cells *in vivo* such as silicon carbide nanoparticles, RESV-loaded PLGA nanoparticles (RESV-NPs), organic semiconducting polymer (OSP)-based nanoprobes, and others.^27−32^ But these studies, similar to our earlier work, have mostly focused on the spatial tracking of stem cells. This study advances stem cell monitoring by additionally providing functional information about the apoptotic status of injected stem cells *in vivo*.

Ultrasound guided photoacoustic (US/PA) imaging has shown great potential for functional imaging coupled with deep tissue anatomical information and fine spatial resolution.^33−35^ Its noninvasive and nonionizing imaging capabilities, familiarity of equipment to clinical staff, small footprint, portability, and relatively low cost make US/PA imaging attractive for clinical applications, including stem cell therapy.^36−38^ US imaging provides excellent anatomical images of tissue with high spatial resolution, while complementary PA imaging can convey functional information with high contrast, resolution, imaging depth, and sensitivity. PA signals are generated by optical absorption of endogenous or exogenous contrast agents.^39^ Thermal deposition rapidly expands the surrounding tissue to generate an acoustic signal.^40^ By using stimuli-responsive exogenous contrast agents, we can significantly enhance PA signals and thereby monitor cellular and molecular events in tissues or microenvironments over time.^41^ Together US/PA imaging can visualize and monitor a highly orchestrated set of events in tissues or microenvironments over time.

Gold nanospheres (AuNSs) have long been considered excellent exogenous PA contrast agents because of their strong optical absorption, biocompatibility, and surface tunability.^41^ However, the use of single AuNSs for *in vivo* US/PA imaging applications has not been successful due to their peak optical absorption occurring at short wavelengths, where light penetration depth is extremely limited and the optical absorption of endogenous absorbers (e.g., hemoglobin, melanin) generates significant background signals.^42^ Instead, clustering of multiple AuNSs with consequent plasmon coupling between nanospheres induces redshift and broadening of optical absorption, allowing the detection of PA signals in the near-infrared (NIR) window at 650–900 nm, where tissue penetration is better due to lower scattering and the optical absorption of biological tissues is minimal.^37,43^

Here we report an approach for longitudinal tracking of stem cells both spatially and functionally using real-time US/PA imaging coupled with a nanoprobe to monitor the apoptosis of injected stem cells. Specifically, we designed a AuNS-based nanoprobe responsive to caspase-3, a major executioner protein in mammalian cell apoptosis.^44^ Caspase-3 is formed by cleavage of procaspase-3 via caspase8/9 or other pro-apoptotic stimuli and subsequent dimerization, and its production is a critical point of no return in apoptosis.^45^ We focused on caspase-3, since the primary mode of cell death in transplanted stem cells is anoikis, a form of apoptosis in which both the extrinsic and the intrinsic pathways culminate through activation of caspase-3.^16,44−47^

**Figure 1:**
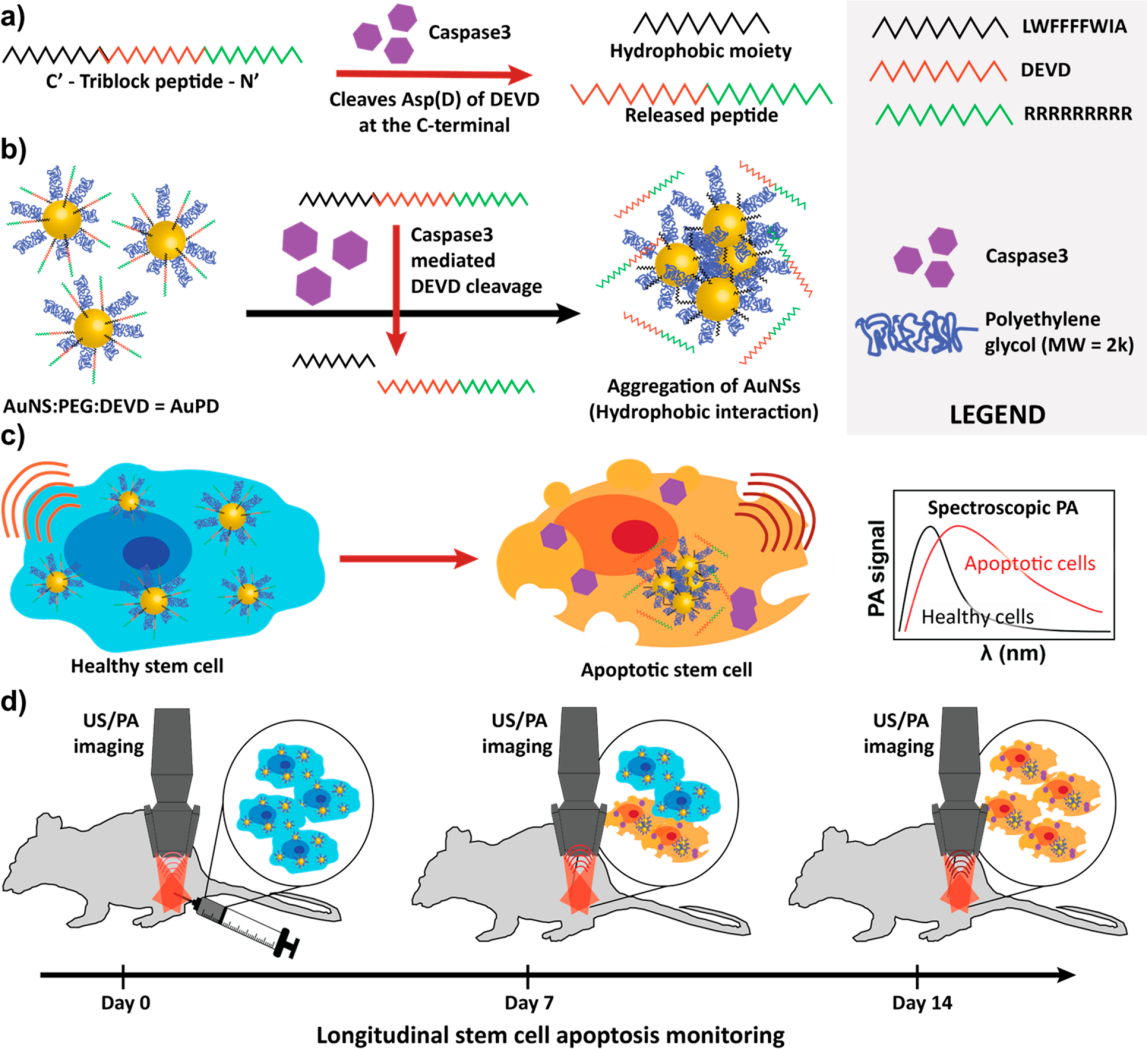
Schematic illustration of caspase-3-responsive nanoprobe (AuPD) components and mechanism of action. (a) Triblock peptide sequence and its cleavage in the presence of caspase-3. (b) Aggregation of AuPDs upon the cleavage of the DEVD moiety and consequent exposure of the hydrophobic surface in aqueous solution. (c) The expected spectroscopic change in the PA signal in healthy cells (left) and apoptotic cells (right), in which AuPD aggregation occurs. Red arcs indicate PA signal upon laser irradiation. (d) Longitudinal noninvasive *in vivo* monitoring of stem cell location and apoptosis using ultrasound-guided spectroscopic photoacoustic (US/PA) imaging.

Our nanoprobe, which we term AuPD, consists of three main components (Figure 1): (1) a triblock peptide containing a cell-penetrating peptide sequence (oligo arginine; R9), a caspase-3-cleavable sequence (DEVD), and a hydrophobic amino acid sequence ending with cysteine (AIWFFFFWLCC); (2) plasmonic AuNS as a core material; and (3) polyethylene glycol (PEG, MW: 2k) as a nanoparticle-stabilizing agent.^48^ The R9 segment in the triblock peptide enables efficient cellular internalization of the nanoprobe, i.e., efficient labeling of cells, facilitating spatial tracking during therapy.^49^ If/when labeled cells undergo apoptosis, the DEVD moiety is selectively cleaved by caspase-3, thereby inducing AuNS aggregation and causing an increase in PA contrast at the NIR window.^50^ Using our specialized nanoprobe, we carried out a series of studies where we used US/PA to spatially and functionally track human adipose-derived mesenchymal stem cells (hADMSCs) *in vitro* and *in vivo* in an ischemic muscular atrophy disease model.

## Results and Discussion

### Synthesis and Characterization of AuPD for PA Imaging

**Figure 2:**
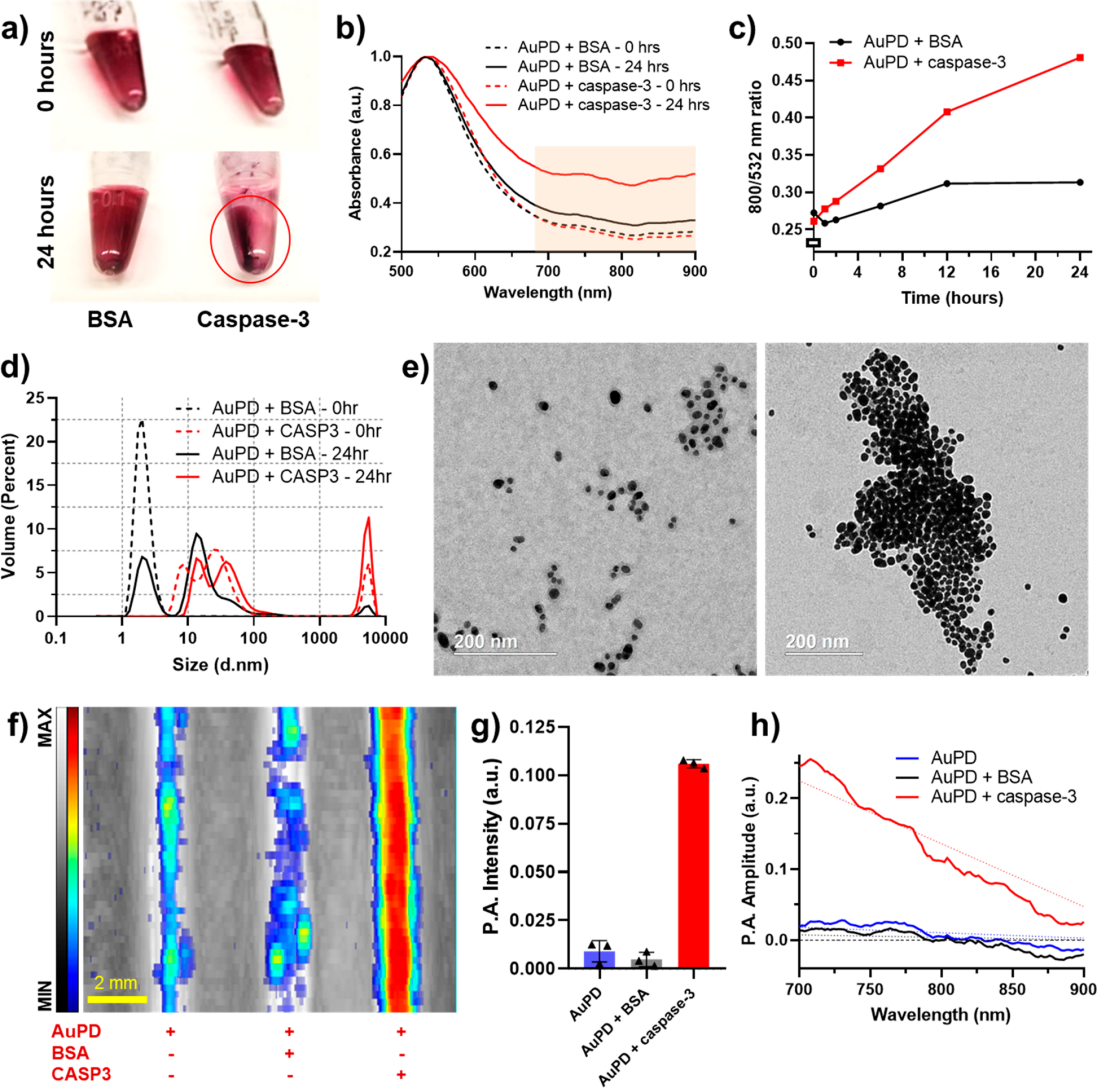
Characterization of the caspase-3-responsive behavior of AuPD. (a) AuPD (2 nM) was incubated with bovine serum albumin, BSA (control, left tubes), and caspase-3 (5 μg/mL, right tubes) for 0 h (top) and 24 h (bottom). Aggregation and colorimetric changes are clearly visible in the presence of caspase-3. (b) UV–vis spectra of AuPD incubated with BSA (control, black) and caspase-3 (5 μg/mL, red) for 0 h (dashed lines) and 24 h (solid lines) show a change in absorbance upon AuPD aggregation. The shaded region represents the NIR-I optical window. (c) Nanoparticle aggregation kinetics were observed by plotting the optical absorption of AuPD nanoparticles (ratio of absorbance at 800 nm/532 nm) vs incubation time. (d) Volume-weighted hydrodynamic size distributions of AuPD nanoparticles incubated with BSA (control) and caspase-3 (2 μg/mL and 5 μg/mL) at 0 and 48 h, showing increased aggregation upon incubation with caspase-3, with only modest aggregation in the presence of control BSA. Plotted data are an average of *n* = 3 technical replicates. T0 and T48 refer to measurements made at time of initiation of incubation and 48 h after incubation, respectively. (e) TEM images of AuPD incubated for 24 h with BSA (control, left) and caspase-3 (right) confirm this aggregation. (f) US/PA overlay image of tube phantom containing solutions of AuPD only (left), AuPD with BSA (middle), and AuPD with caspase-3 (CASP3, 5 μg/mL, right) after 24 h of incubation (wavelength = 800 nm; *n* = 3 replicates). Note that the PA signal of the AuPD only solution is sufficient for cell tracking and is significantly enhanced in the presence of capsase-3. The US image is shown in a grayscale map (a.u.), and the PA image is shown in a color scale map (a.u.). Scale bar is 2 mm. (g) Quantitative PA amplitude of AuPD incubated for 24 h with BSA or activated caspase-3 in the tube phantom (wavelength = 800 nm, *n* = 3 replicates measured 3 times) shows a 22-fold increase in AuPD PA signal in the presence of caspase-3 vs in the presence of BSA. (h) Spectroscopic PA amplitude at 700–900 nm of AuPD incubated with BSA or activated caspase-3 for 24 h (*n* = 3) shows the change in PA spectral slope in the presence of caspase-3 vs in the presence of BSA, confirming the potential of AuPD as a nanosensor for cell apoptosis. The dotted lines represent best-fit linear regressions of PA amplitude on wavelength for all samples. The dashed line represents the case of PA amplitude = 0. In panels (d) and (g), dots show individual data points, and error bars are standard deviations, which are not visible for some conditions due to high repeatability. In panels (g) and (h), the plotted quantities are amplitudes after subtracting the amplitude from a water blank, correcting for any laser energy drift.

We first optimized the AuPD design by testing a variety of ratios of AuNSs, PEG chains, and triblock peptides to achieve high nanoparticle stability under physiological conditions, low nonspecific aggregation, and a sensitive and specific aggregation response to caspase-3. Transmission electron microscopy (TEM) images and dynamic light scattering (DLS) measurements showed successful modification of the surfaces of AuNSs with no major morphological changes or severe aggregation of AuPEG and AuPD (Figure S1a,b). In AuPD, however, modest AuNS clustering occurred due to the hydrophobic component of the triblock peptide, which resulted in slightly increased optical absorption in the NIR window (Figure S1b,c). Next, caspase-3-responsive aggregation of AuPD was confirmed through colorimetric observation and UV–vis spectrometry in an aqueous solution. In the presence of caspase-3, there was significant aggregation of AuPD due to the cleavage of the DEVD peptide and subsequent hydrophobic nanoparticle aggregation, which was absent under control conditions (incubation with bovine serum albumin [BSA]) (Figure 2a,b). The caspase-3-sensitive aggregation kinetics of AuPD were also studied, with a ∼54% increase in the optical absorption ratio at 800 nm vs 532 nm within 24 h (Figure 2c) of caspase-3 exposure. Volume-weighted hydrodynamic size distributions showed far greater aggregation of AuPD in the presence of caspase-3 versus BSA, which was confirmed by TEM imaging (Figure 2d,e). These characterization results, notably the significant redshift and increase in the optical absorption spectrum due to plasmon coupling in the NIR region of 680–970 nm, supported further testing of the AuPD nanosensor as a PA contrast agent for tracking injected stem cells and for sensing the presence of caspase-3.

We therefore went on to demonstrate the AuPD nanosensor for PA imaging using a polyethylene tube phantom containing AuPD and its precursors in solution (Figure S1d). In the absence of caspase-3, there was no qualitative or quantitative difference in PA signal between AuNS and AuPEG, while AuPD showed a slight increase in PA signal, which was attributed to minimal nonspecific clustering of the particles (Figure S1e). This clustering and the consequent small PA signal increase in the NIR window were consistent with the ability to spatially track viable stem cells with negligible intracellular caspase-3. Next, caspase-3-responsive PA signal amplification was tested in the tube phantom by incubating AuPD with BSA (control) or caspase-3 at different concentrations and for a range of incubation times at 37 °C (Figures 2f and S2). In all cases, we saw similar signals from the AuPD only and AuPD incubated with BSA groups, while AuPD incubated with caspase-3 showed a significantly stronger PA signal (Figure 2f). Quantitatively, we observed a 22-fold increase in the amplitude of the PA signal from AuPD at 800 nm 24 h after the addition of caspase-3 vs BSA control (Figure 2g). Spectroscopic PA analysis from 680 to 900 nm further demonstrated a caspase-3-responsive redshift in optical absorption, corresponding to a 25.7-fold increase in the slope of the graph of PA amplitude vs wavelength of the AuPD nanoprobe incubated with caspase-3 vs BSA control (Figures 2h and S2c). Finally, photostability of the AuPD was tested using >1000 laser pulses at different wavelengths to confirm its utility as a longitudinal PA contrast agent (Figure S3). There was no change in the PA amplitude or the PA spectrum over time, which establishes the photostability of our nanosensor for long-term monitoring.

### Labeling hADMSCs with AuPD for Spatial Tracking

**Figure 3:**
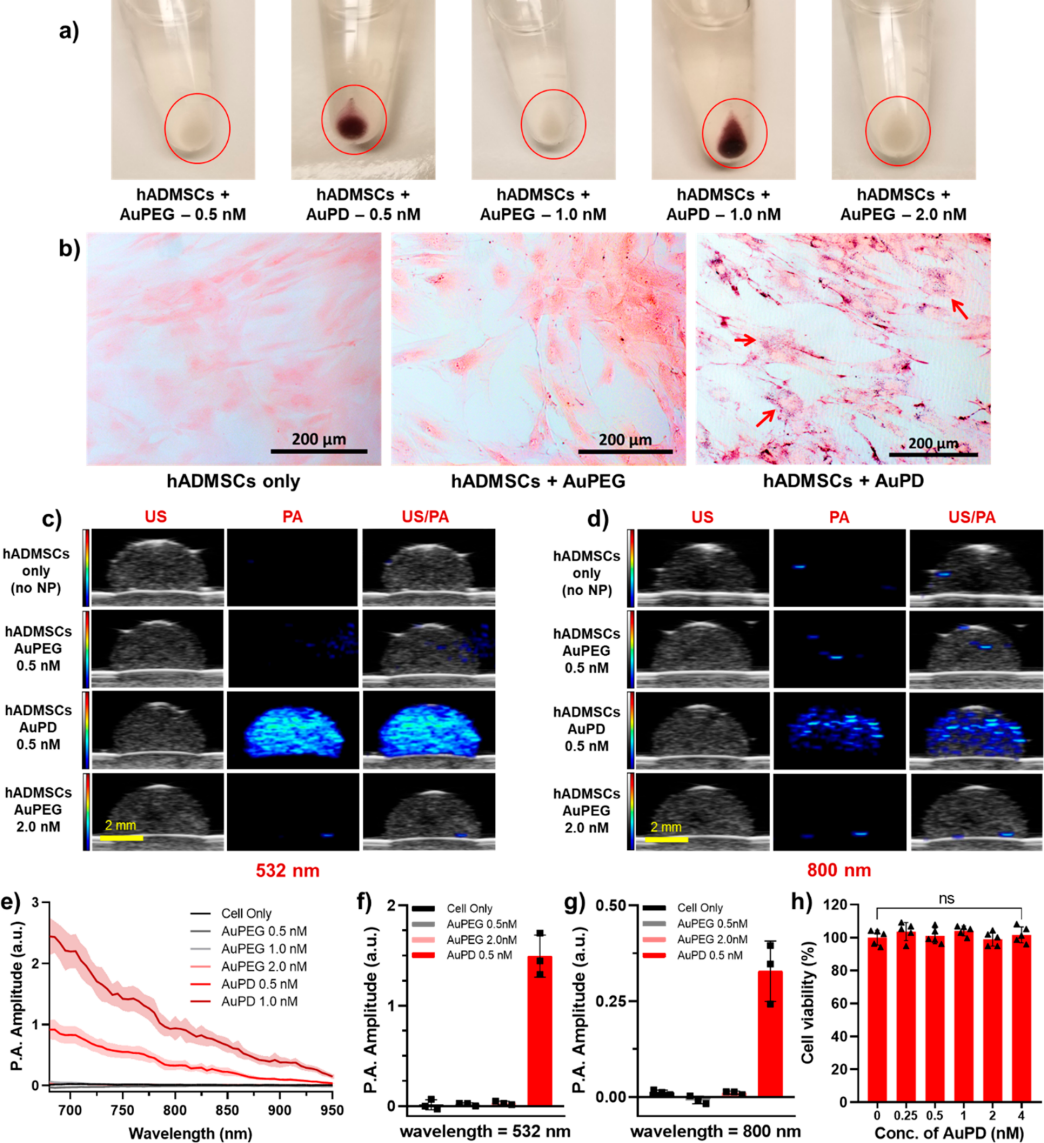
Characterization of the cellular uptake of AuPD. (a) Images of human adipose derived mesenchymal stem cells (hADMSCs) labeled with a nanoprobe without triblock peptide (AuPEG) at 0.5, 1.0, and 2.0 nM and with the AuPD nanosensor at 0.5 and 1.0 nM. (b) Bright-field images of hADMSCs only (left), cells labeled with AuPEG (middle, 0.5 nM), and cells labeled with AuPD (0.5 nM, right). Red arrows point to purple speckles indicating AuPDs (scale bar = 200 μm) which have been internalized by the cell and are present overlaid onto the pink eosin-stained cytoplasm. (c, d) US (left column), PA (middle column), and US/PA (right column) images of a gelatin dome phantom containing hADMSCs only (top row), cells labeled with AuPEG at 0.5 nM (second row) and 2.0 nM (fourth row), and AuPD nanoprobe at 0.5 nM (third row) imaged at (c) 532 nm and (d) 800 nm wavelengths (scale bar = 2 mm). There is no PA signal from control AuPEG-labeled hADMSCs due to minimal cellular uptake, in contrast to the substantial PA signal in the AuPD-labeled hADMSCs. The US images are shown in grayscale map (a.u.), and the PA images are shown in color scale map (a.u.). (e) Spectroscopic PA amplitude of hADMSCs only, AuPEG-labeled hADMSCs (0.5, 1.0, 2.0 nM), and AuPD-labeled hADMSCs (0.5, 1.0 nM) at 680–950 nm (*n* = 3; solid line is mean, while shaded regions represent the standard deviation). (f, g) Quantitative PA amplitude of hADMSCs only, AuPEG-labeled cells (0.5 nM, 2.0 nM), and AuPD-labeled cells (0.5 nM) at (f) 532 nm and (g) 800 nm from the dome phantom (*n* = 3). From panels (e), (f), and (g) we can see that AuPEG-labeled cells demonstrate no PA signal due to minimal nanoparticle uptake, while AuPD-labeled cells show strong PA signal, consistent with excellent nanoparticle uptake. Even cells incubated with a low 0.5 nM AuPD concentration displayed strong PA signal, motivating selection of this concentration for future cell labeling. (h) Viability of hADMSC cells labeled with different concentrations of AuPD showing no significant cell death at up to 8 times the working concentration (0, 0.25, 0.50, 1.0, 2.0, and 4.0 nM; *n* = 5 each; ns: *p* > 0.05 by unpaired *t* test across control vs each of the AuPD concentrations).

To spatially and functionally track injected stem cells, we must be able to efficiently label the cells using a AuPD nanoprobe. For this purpose, we incorporated an R9 segment in the triblock peptide to enable efficient cellular uptake/labeling. We tested this functionality by culturing and labeling hADMSCs with different concentrations of AuPEG and AuPD for 24 h. A deep purple pellet demonstrated efficient labeling of cells using AuPD as compared to the essentially colorless AuPEG-incubated cell pellet (Figure 3a). Bright-field (BF) imaging further confirmed enhanced stem cell uptake of AuPD as compared to AuPEG, as indicated by the purple speckles co-localized with the pink eosin-stained cytoplasm (Figure 3b, red arrows). Next, US/PA imaging was conducted using a tissue mimicking 8% gelatin phantom with dome-shaped inclusions containing identical concentrations of cells (500 cells/μL) labeled with AuPEG or AuPD at different concentrations (Figure 3c). It was observed that the US signal remained similar over all conditions (left column, grayscale), while AuPD-labeled cells had noticeably greater PA signal than the AuPEG-labeled cells at single wavelengths, 532, 700, 750, and 800 nm (middle column, color scale), and over the spectral range of 680–950 nm (Figures 3c–g, S4, and S5). Based on these results, an AuPD concentration of 0.5 nM was selected as optimal for cell labeling. At this concentration, the PA signal at 800 nm was sufficient for stem cell spatial tracking in the absence of apoptosis (Figure 3c,d), but it was still low enough to allow for an obvious signal increase (without saturation) in the presence of caspase-3 and subsequent AuPD aggregation (see below).

In addition, cell–nanosensor interactions were studied to evaluate the effect of AuPD labeling on stem cells. Although numerous reports have characterized AuNP-based nanoprobes as nontoxic,^51−53^ toxicity evaluation of the nanoprobe was conducted to ensure there was no false-positive signal induced by nanoprobe-triggered cell death. Based on the standard MTT cell viability assay, no cell death was observed even at 4.0 nM AuPD labeling, which is 8× higher than the selected working concentration (Figure 3h). Further, an Annexin-V cell apoptosis assay confirmed the negligible effect of AuPD on cell apoptosis, exhibiting no qualitative and quantitative difference in the Annexin signal in both unlabeled and AuPD-labeled cells (Figure S6). To assess the functional impact of AuPD labeling on MSC multipotency, a tridifferentiation assay was employed. Results from the tridifferentiation assay revealed that both the AuPD-labeled and AuPD-negative (unlabeled) stem cells exhibited similar differentiation capabilities, with no significant differences observed (Figure S7). Adipocyte differentiation remained unaffected by the presence of AuPD nanosensors, as evidenced by the presence of red lipid vacuoles stained with Oil Red O (Figure S7c,d). The development of a deep sky-blue marbling pattern of polysaccharides stained by Alcian blue indicated successful chondrocyte differentiation in both the AuPD-labeled and unlabeled cells (Figure S7g,h). Additionally, increased mineral deposition, visualized by Alizarin Red staining, was observed in the osteogenic differentiation experiments, depicted as deep orange-red patterning and circular deposits (Figure S7k,l). These characteristics were observed in both the AuPD-labeled and unlabeled cells, demonstrating similar capacities for differentiation. Importantly, the presence of AuPD nanosensors per se did not induce any unintended differentiation, as confirmed by labeling of AuPD-containing cells grown under control (noninducing) conditions that did not demonstrate differentiation (Figure S7a,b,e,f,i,j). These findings indicate that AuPD labeling does not compromise the differentiation capacity of hADMSCs, nor does it lead to unintended differentiation outcomes. To determine whether AuPD labeling affected several molecular phenotypic markers of hADMSCs, we immunolabeled cells for CD90 and CD14 surface markers using FITC-conjugated antihuman antibodies (Figure S8), since CD90 and CD14 are known positive and negative markers for hADMSCs, respectively.^54,55^ We observed the expected high level of CD90 labeling and almost negligible CD14 labeling on the surface of AuPD-negative (control) cells. Importantly we also found that AuPD labeling did not change these patterns, both qualitatively (Figure S8a–d) and by quantification of fluorescent signals (Figure S8e,f), indicating that AuPD labeling did not alter several characteristic molecular phenotypes of our cells.

### Detecting Intracellular Caspase-3 Activity *in Vitro*

**Figure 4:**
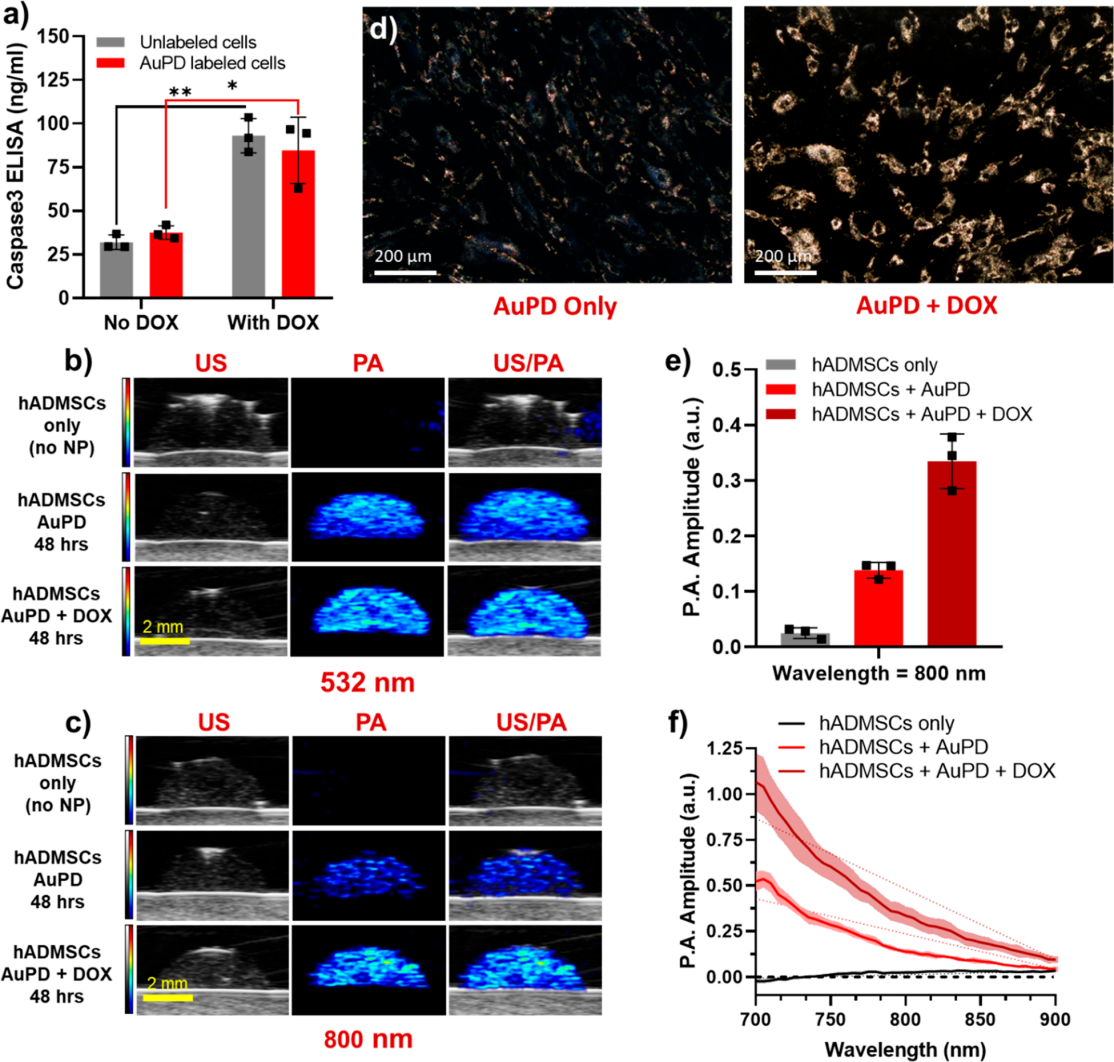
Characterization of the activity of the AuPD nanosensor *in vitro*. (a) Caspase-3 levels measured by ELISA in AuPD-labeled and unlabeled hADMSCs with/without 20 μM DOX (*n* = 3, *p < 0.05, **p < 0.01 by unpaired *t* test). There are significantly greater caspase-3 levels in cells incubated with DOX vs in those without DOX. (b, c) US (left column, grayscale [a.u.]), PA (middle column, color scale [a.u.]), and US/PA overlay (right column) images of a gelatin dome phantom containing hADMSCs only (top row), AuPD-labeled cells without DOX (middle row), and AuPD-labeled hADMSCs with 20 μM DOX (bottom row) imaged at (b) 532 nm and (c) 800 nm wavelength after 48 h (scale bar = 2 mm). Note that the PA signal at 532 nm is similar with/without DOX but increases significantly in amplitude at 800 nm in DOX-treated cells. (d) Dark-field images of AuPD-labeled hADMSCs without DOX (left) and incubated with 20 μM DOX (right) at 24 h (scale bar = 200 μm). Cell apoptosis and resulting aggregation of AuPD inside hADMSCs is clearly visible with DOX incubation vs without. (e) Quantitative PA amplitude at 800 nm from the dome phantom containing hADMSCs only and from AuPD-labeled cells with and without 20 μM DOX incubation (48 h, *n* = 3). There is a 2.4-fold increase in PA signal at 800 nm in AuPD-labeled hADMSCs exposed to DOX vs without DOX. (f) Spectroscopic PA amplitude of hADMSCs only and of AuPD-labeled hADMSCs with and without 20 μM DOX incubation at 700–900 nm (48 h, *n* = 3). Solid lines indicate mean spectra; shaded regions represent standard deviations. The dotted lines represent the best fit linear regressions of PA amplitude on wavelength for all samples.

Next, we validated the capability of our AuPD nanosensor to detect intracellular caspase-3. To induce caspase-3 in hADMSCs, 0.5 nM AuPD-labeled cells were incubated with 20 μM of the cytotoxic agent doxorubicin (DOX). We first used ELISA to quantify the amount of caspase-3 in both labeled and unlabeled cells treated with/without DOX. Consistent with the Annexin-V staining described above, we observed no significant difference in caspase-3 levels between the unlabeled and the labeled cell groups; however, there were significantly greater caspase-3 levels in cells treated with DOX (Figures 4a and S9). Next, the AuPD-labeled hADMSCs treated with/without DOX were harvested and observed using BF and dark-field (DF) microscopy and with PA imaging. BF microscopy images showed very clear and significant aggregation of AuPD in DOX-treated hADMSCs vs no DOX control cells (Figure S10). DF microscopy images also confirmed the aggregation of AuPD in DOX-treated apoptotic cells, as shown by the much stronger scattering signals vs labeled cells without DOX or other controls (Figures 4d and S11). As before, the associated PA signal was acquired from a tissue mimicking 8% gelatin dome phantom containing equal concentrations of AuPD-labeled cells (500 cells/μL) with/without DOX treatment. There was no significant PA signal difference observed at 532 nm, which was expected since peak optical absorption of unaggregated AuPD occurs near 532 nm. However, at wavelengths in the NIR window, including 700, 750, and 800 nm, the PA signal was significantly enhanced in DOX-treated stem cells, an effect we ascribe to DOX-triggered apoptosis, AuPD aggregation, plasmon coupling, and ultimately PA spectral shifts and signal increases at longer wavelengths (Figures 4b,c and S12). Quantitatively, the average PA amplitude from a dome phantom demonstrated no significant difference between AuPD-labeled cells with and without DOX treatment at 532 nm but showed a 2.4-fold increase in signal amplitude at 800 nm in DOX-treated cells vs no-DOX controls (Figure 4e). Average PA signals at 700 and 750 nm also displayed similar trends (Figure S12c). Spectroscopic PA analysis confirmed these results, showing both an increase in amplitude and a 2-fold increase in spectral slope from 700 to 900 nm between AuPD-labeled cells with and without DOX incubated for 48 h due to the aggregation of AuPD in response to activated caspase-3 (Figure 4f). These results clearly demonstrate the ability of our AuPD nanoprobe to detect stem cell location and apoptosis using US/PA imaging at an 800 nm wavelength.

### Longitudinal Monitoring of Stem Cell Apoptosis *in Vivo*

Lastly, we sought to validate our AuPD nanosensor in a preclinical US/PA imaging scenario by demonstrating the spatial tracking of injected stem cells and apoptosis monitoring in an animal model. Specifically, we employed the widely used unilateral hindlimb muscle atrophy model in mice, in which ischemia is induced by ligation of the femoral artery and vein.^56,57^ AuPD-labeled hADMSCs suspended in Matrigel were injected intramuscularly immediately after ischemia-inducing surgery in the hindlimb downstream from the ligated section. In this model, we did not use the contralateral limb due to concerns about a systemic inflammatory response. Instead, we used two control groups: (1) a “nonischemic” group received an injection of AuPD-labeled hADMSCs but no ischemia-inducing surgery and (2) an “unlabeled” group of mice received an injection of Matrigel only or unlabeled hADMSCs suspended in Matrigel. The latter group was further subdivided: some animals received ischemia-inducing surgery, while others did not (Figures S13 and S14). We hypothesized that the ischemic model receiving AuPD-labeled stem cells would demonstrate significantly greater PA signal and PA spectral slope in the NIR wavelength, i.e., 700–900 nm, due to stem cell apoptosis vs the nonischemic model receiving AuPD-labeled stem cells.

**Figure 5:**
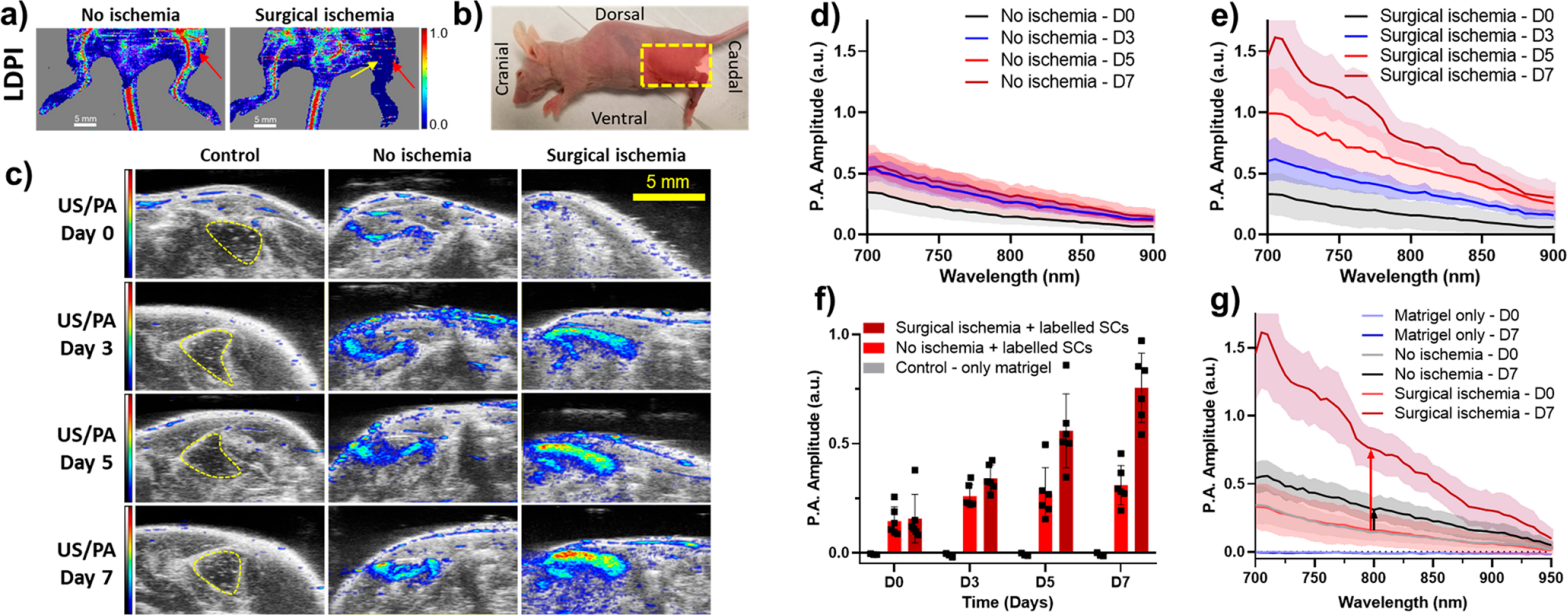
Characterization of the *in vivo* activity of the AuPD nanosensor. (a) Representative laser Doppler perfusion images (LDPI) from the hindlimb ischemia mouse model and control animals (scale bar = 5 mm). The color map shows blood flow (laser Doppler perfusion index, arbitrary units, with warm colors indicating greater perfusion). The yellow arrow indicates the site of ligation of the femoral artery and vein. The red arrows indicate the site of stem cell injections. The nonischemic mouse clearly shows blood flow in the intact femoral artery in the hindlimb region, whereas minimal blood flow is visible in the surgical ischemia model. (b) The orientation of the mouse during US/PA imaging. Yellow box: imaging window where the transducer was placed. (c) Representative US/PA images (single slices) of control (Matrigel injection only; left column), nonischemic (middle column), and surgical ischemia (right column) limbs at days 0 to 7 imaged at 800 nm. No PA signal is visible in the Matrigel-only control, where the yellow dashed lines indicate the Matrigel injection location. PA signal is evident in the no ischemia and surgical ischemia models, both of which were injected with AuPD-labeled hADMSCs. We observe gradual PA signal increase in the no ischemia model due to mild apoptosis vs significant PA signal increase in the surgical ischemia model, consistent with severe apoptosis. The US images are shown in a grayscale map (a.u.), and the PA images are shown in a color map (a.u.). Scale bar is 5 mm. (d, e) PA amplitude spectrograms from AuPD-labeled hADMSCs injected intramuscularly, measured at 700–900 nm longitudinally (days: D0, D3, D5, and D7) for the (d) nonischemic model and (e) surgical ischemia model (*n* = 6 animals/group). (f) Quantitative PA amplitude from Matrigel-only (control) and AuPD-labeled hADMSCs injected intramuscularly, measured at 800 nm longitudinally (days: D0, D3, D5, and D7) for the nonischemic and the surgical ischemia models (*n* = 3 animals/control group and *n* = 6 animals/experimental group). (g) Spectroscopic PA amplitude measured in animals injected with Matrigel only (control) and AuPD-labeled hADMSCs, measured at 700–950 nm longitudinally (days: D0, D7, and D14) for the nonischemic and the surgical ischemia models (*n* = 3 animals/control and *n* = 6 animals/experimental group). Panels (f) and (g) show the ability to detect different rates of stem cell apoptosis in ischemic vs nonischemic models by using US/PA imaging of AuPD-labeled stem cells. In panels (d), (e), and (g), solid lines are means and shaded regions show standard deviations.

Following previous methods, the femoral artery and vein were ligated and excised to create a local hypoxic environment, ultimately resulting in muscle deterioration at and distal to the site of surgery. The presence of hindlimb ischemia was first verified using laser Doppler perfusion imaging (LDPI), showing substantially reduced blood flow in ischemic (operated) legs (Figure 5a,b). The US/PA signal from injected cells was monitored longitudinally at days 0, 3, 5, and 7 in mice and additionally at days 10, 12, and 14 in the unlabeled and no ischemia models. At 800 nm, negligible PA signal was observed from the Matrigel-only control limbs from day 0 to day 7 (Figure 5c, left column). The PA signal from AuPD-labeled hADMSCs increased from days 0 to 7 in all conditions due to stem cell apoptosis, but a significantly greater signal was observed in the ischemic limbs (Figure 5c, right column) vs in the nonischemia model (Figure 5c, middle column). Spectroscopic PA analysis confirmed that there was significantly greater signal in the ischemic limbs vs in the nonischemia model (Figure 5d,e and Video S1). Quantitative and spectroscopic PA amplitude at 800 nm on day 7 showed a 4.8-fold increase in signal in the ischemia model, which was greater than the 2.1-fold increase observed in the nonischemia limbs (Figure 5f,g).

**Figure 6:**
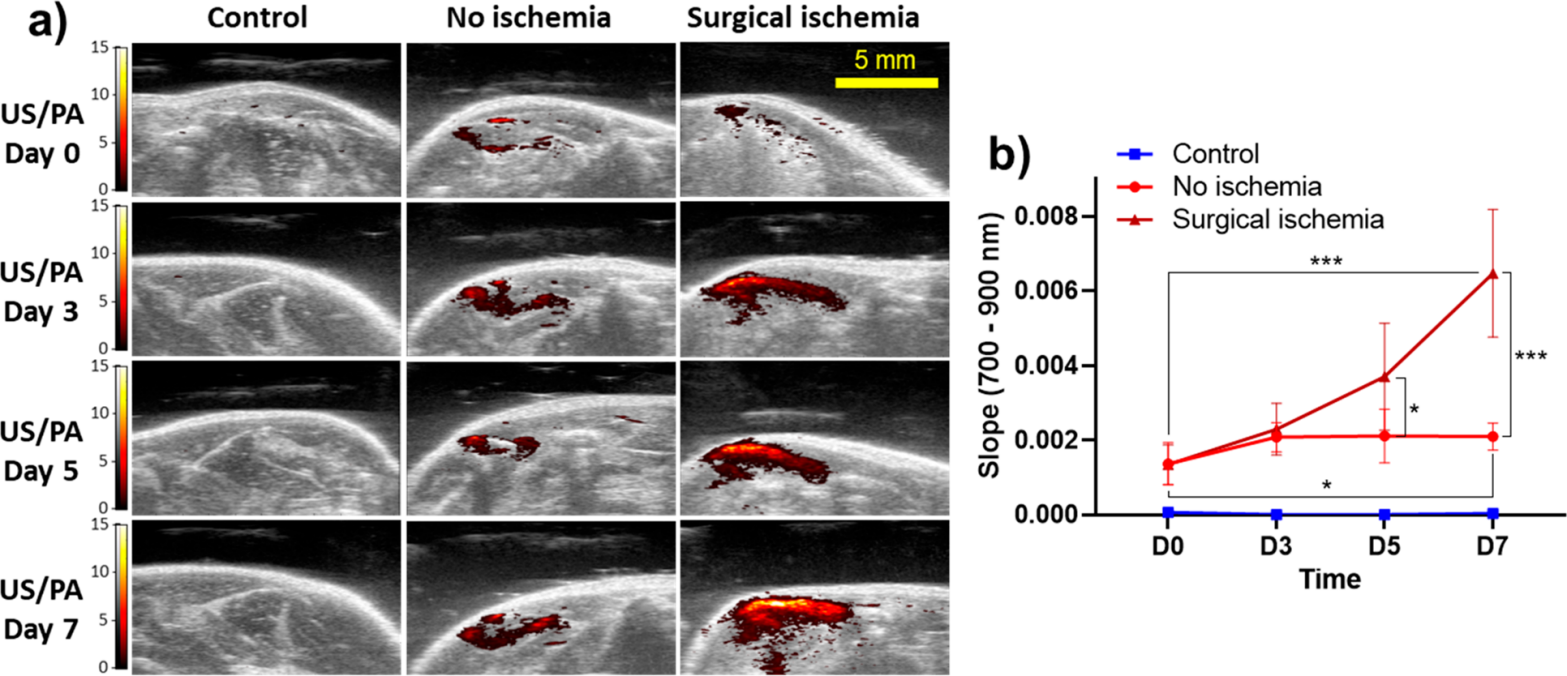
Slope analysis for the *in vivo* activity of AuPD nanosensors. (a) Slope analysis images (see description in the text) from 700 to 900 nm of Matrigel-only control (left column), AuPD-labeled hADMSCs injected intramuscularly in the nonischemic model (middle column), and AuPD-labeled hADMSCs in a surgical ischemia model (right column) from days 0 to 7 (scale bar = 5 mm). We see similar trends with a slight change in slope in the no ischemia model but a substantial increase in the slope in the surgical ischemia, showing significantly greater injected stem cell apoptosis. (b) Slopes from linear regression of PA spectra on wavelength (700–900 nm) in Matrigel-only controls (D0 to D7, *n* = 3) and AuPD-labeled hADMSCs in the nonischemic and the surgical ischemia models vs time (days D0 to D7). Average PA values were calculated from a region of interest at each wavelength from 700 to 900 nm and used to perform a least-squares linear regression for each (*n* = 3 animals for control and *n* = 6 for experimental). Plotted values are means; error bars are standard deviations. Data were analyzed by two-factor ANOVA with replication on days and ischemic condition. The days, ischemic condition, and their interaction were significant (*p*-values 1.869 × 10^–8^, 1.041 × 10^–6^, 1.822 × 10^–6^, respectively). This was followed by *post hoc* testing (Dunnett’s test, *p < 0.05, **p < 0.01, ***p < 0.001).

The data shown in Figure 5 are promising, but the average PA signal readout is not an ideal outcome measure, since it is amplitude dependent, which can fluctuate due to various AuPD aggregation-independent factors such as laser energy, fluence, location (depth) of the nanosensor, and the concentration of the nanosensor. We reasoned that the spectral slope (slope of linear regression of PA amplitude on wavelengths of 700–900 nm) could overcome some of these drawbacks, while also helping to eliminate noise with no defined slope, such as residual signals from the skin. We therefore created “slope maps” by processing the *in vivo* images in Figure 5b to compute a spectral slope (as defined above) for each pixel (Figures 6a and S15). We observed that the temporal change in the spectral slope was significantly greater for the surgical ischemia model (4.8-fold increase from days 0 to 7) vs in the nonischemic model (1.5-fold increase over the same period), presumably due to greater aggregation of the nanosensor upon caspase-3 activation (Figures 6b and S15). This result indicates the ability of our AuPD nanosensor to detect the kinetics of stem cell apoptosis *in vivo*. Additionally, using a translational motor, three-dimensional (3D) US/PA imaging was performed, with US showing the anatomy of the hindlimb region and the PA signal describing the distribution and spread of the labeled stem cells in 3D over time and the distribution of apoptosis in 3D over time (Figures S16, S17 and Video S2). Histology of the extracted hindlimb tissue confirmed the presence of tissue damage and AuPD-labeled stem cells in the hindlimbs of the mice (Figure S18). We conclude, as demonstrated in an *in vivo* mouse model of apoptosis, our nanosensor has utility for longitudinal and noninvasive tracking and apoptosis monitoring of injected stem cells.

Compared to existing *in vivo* apoptosis sensors,^38^ our US/PA approach has many advantages. Previous work in this area involved the design of fluorescent probes which, although very sensitive, are limited due to poor tissue penetration of visible light and photobleaching concerns, consequently restricting their suitability for longitudinal *in vivo* imaging, and MRI- and PET-based probes, which allow deep tissue imaging, but do not possess real-time observational capabilities or peri-procedural guidance.^58−63^ There have been a variety of nanoparticle-based PA contrast agents to track MSC location, but none of them provide any function information about stem cell status.^27,30−33,64^ Recently, contrast agents have been developed that use PA imaging to monitor stem cell apoptosis using reactive oxygen species (ROS) as an indicator of apoptosis.^59,65^ However, ROS is abundantly present during degenerative and atrophic disease conditions, and thus ROS-based probes can lead to significant false-positive signals from neighboring cell death, resulting in low specificity of determining apoptosis of injected stem cells.^66^ Furthermore, this probe also employed a PA signal turn-off mechanism which does not distinguish between increased apoptosis and cell escape/relocation due to the PA signal drop in both situations. Our AuPD nanosensor improves on these existing approaches by using caspase-3 as the cell apoptosis biomarker, a specific intracellular factor that is highly localized within the labeled stem cells being monitored.^67^ This allows us to utilize a “turn-on” biosensing method, which, when coupled with a PA signal shift to detect apoptosis, eliminates the confounding effects of cell escape and relocation.

Our nanosensor has enormous potential for the advancement of preclinical stem cell research by allowing noninvasive longitudinal monitoring of stem cell location and apoptosis *in vivo*, e.g., during studies of pharmacological agents and/or biomaterials delivered with the stem cells. We also anticipate that our sensor will reduce animal use in stem cell research due to the decrease in end-point histology, staining, and assay-based examinations and will improve research throughput with reduced *in vivo* study timelines thanks to longitudinal monitoring. Further, the nanosensor can also be used to study the pathophysiology of disease models and determine kinetics of cell apoptosis during degenerative disease progression. In a clinical setting, physicians could eventually use AuPD and US/PA imaging to perform guided delivery of stem cells, then longitudinally monitor the retention of stem cells after injection to provide better patient outcomes, for example, using this information to determine optimal times and dosages for stem cell reinjection. This approach could also be used to study the effects of new procedures or pharmacological interventions to improve implanted stem cell viability in a clinical setting.

However, we recognize that there are important considerations for using US/PA coupled with AuPD in a clinical setting. Most importantly, PA imaging has inherent limitations regarding background noise from tissue, light scattering, and a finite depth profile that together lead to significant signal reduction in deep tissues. Thus, monitoring deep tissue areas will require a modified PA imaging system, including PA tomographic systems or possibly an invasive ultrasound and photoacoustic imaging probe. An important consideration during cell nanoengineering is the fate of the nanoparticles after cell apoptosis or release of nanoparticles through exocytosis, which could lead to false-positive signals. To mitigate such effects, our lab has previously used a dual-nanoparticle system to perform multiplex PA imaging which, through the use of preferential labeling of nonstem phagocytotic cells vs transplanted stem cells *invivo* along with information about the spatial co-localization of the signal, will result in minimal false-positive signals.^28^ Additionally, the DEVD peptide is also cleavable by other caspases, primarily caspase-7, which is another effector enzyme and plays an important role in the apoptosis cascade. However, since caspase-3 cleaves DEVD at a 5-fold greater rate than caspase-7 and since caspase-7 is involved in the apoptosis cascade, this cross-talk does not diminish the ability of our sensor to detect apoptosis.^41,68,69^

## Conclusions

AuNSs modified with PEG and triblock peptides were successfully designed, created, and characterized as nanosensors for monitoring the location and apoptotic status of stem cells using US/PA imaging. The triblock peptide had two primary functions: enhancing cellular uptake of AuPDs and acting as a sensitive detector of caspase-3. Characterization showed substantial nanoparticle aggregation in the presence of caspase-3 and significant cellular uptake of AuPD into stem cells, as desired. The concentration of AuPD used for cell labeling was optimized to provide sufficient PA signal for cell tracking while allowing for dramatic signal change upon apoptosis, with no effect on cell viability. Specifically, PA imaging showed both a significant increase in the PA amplitude at 800 nm and a shift in PA spectra in the NIR-I optical window in cells undergoing apoptosis. Motivated by these positive results, we delivered AuPD nanosensor-labeled stem cells in a mouse model of ischemia, showing the ability of our nanoparticles to longitudinally detect caspase-3, a hallmark of apoptosis. Thus, our approach provides a noninvasive and nonadverse imaging method for stem cell functional imaging *in vivo*. Fundamentally, we have designed a sensing mechanism using the change in PA signal caused by the aggregation of AuNSs, which can be used as a platform to design a multitude of sensors through modification of the core materials or surface peptides. Future work will involve modifying the nanosensor core material to facilitate multimodal imaging approaches and increase imaging depth, such as plasmonic magnetic nanoparticles to allow trimodal US/PA/MRI. In addition, through the modification of surface peptides, we hope to design nanosensors responsive to *in vivo* stem cell differentiation and/or unintended malignancy for use in specific regenerative applications such as spinal cord injury, glaucoma, and macular degenerative models. This nanosensor fills a significant current gap in stem cell research by providing an imaging approach that can be used to track and monitor stem cell location and status *in vivo* and thus accelerate preclinical studies.

## Methods and Experimental

### Materials

Gold chloride trihydrate (HAuCl_4_, Sigma-Aldrich), sodium citrate tribasic dihydrate (Sigma-Aldrich), methyl polyethylene glycol thiol (mPEG-SH, MW: 2000, Biochempeg), bovine serum albumin (BSA, Sigma-Aldrich), caspase-3 (CASP3, Abcam), paraformaldehyde (PFA, Sigma-Aldrich), 3-(4,5-dimethylthiazol-2-yl)-2,5-diphenyltetrazolium bromide (MTT, EMD Millipore), dimethyl sulfoxide (DMSO, Sigma-Aldrich), gelatin (MP Biomedicals), silica (0.25 μm diameter, Sigma-Aldrich), potassium cyanide (KCN, Sigma-Aldrich), Dulbecco’s modified Eagle’s medium (DMEM, Cytiva), fetal bovine serum (FBS, Sigma-Aldrich), and penicillin/streptomycin (Sigma-Aldrich) were used in accordance with the manufacturer’s instructions.

### Ultrasound and Photoacoustic Imaging

All US/PA imaging was conducted using a Vevo LAZR 2100 system (Fujifilm VisualSonics, Inc.), which uses a Q-switched Nd:YAG laser with a second-harmonic generator and optical parametric oscillator (laser pulse repetition frequency = 20 Hz, laser pulse duration = 7 ns).

### Synthesis of the AuPD Nanosensor

Citrate-capped 15 nm diameter gold nanospheres were prepared using a bottom-up method as previously reported, involving reduction of gold(III) chloride (HAuCl_4_, 1.47 mM) using sodium citrate tribasic (0.334 M) as both the reducing agent and the stabilizing agent at 80 °C under mild stirring for 30 min.^70^ The ruby-red-colored AuNS colloidal solution was left overnight and then PEGylated using mPEG-SH (MW: 2000) through ligand exchange at a 1:500 molar ratio of AuNSs to mPEG-SH followed by bath sonication for 5 min. Excess PEG was then removed after 24 h by centrifugation (16,100 rcf, 20 min, 3 repeats) to obtain AuPEG. The triblock peptide (GenScript USA, Inc.), consisting of a cell-penetrating peptide sequence (oligo arginine; R9), a caspase-3-cleavable sequence (DEVD), and a hydrophobic amino acid sequence ending with cysteine (AIWFFFFWLCC), was used to functionalize AuPEG at a 1:4000 molar ratio of AuPEG to peptide for 24 h under gentle stirring. The resulting nanosensor was centrifuged to remove excess triblock peptide (16,100 rcf, 20 min, 3 repeats), thereby creating AuNS modified with PEG and the DEVD triblock peptide conjugate (AuPD).

### Characterization of the AuPD Nanosensor

All studies were carried out using BSA as a control and recombinant human active caspase-3 as a target protein. For colorimetric studies, AuPD (2 nM) was dispersed with caspase-3 (5 μg/mL) or BSA (5 μg/mL) and incubated at 37 °C for 24 h to induce aggregation. The optical properties of AuPD with caspase-3 (5 μg/mL) or BSA (5 μg/mL) and incubated at 37 °C for 24 h were measured with a UV–vis–NIR spectrophotometer (Evo 220, Thermo Fisher Scientific) at multiple time points over a 24 h period. Similarly, the volume-weighted hydrodynamic size distributions of AuPD with/without caspase-3 (2 μg/mL, 5 μg/mL) incubated at 37 °C for 24 h was measured by DLS (Zetasizer Nano ZS, Malvern Instruments Ltd.) at multiple time points over a 72 h period. The size, morphology, and aggregation state of the nanosensors were characterized by TEM (HT 7700, Hitachi). The samples were prepared using drop casting, where 10 μL of the nanoparticle solution was dropped on a copper mesh grid, followed by air-drying overnight. The images were analyzed with Gatan Digital Micrograph software.

PA imaging and nanosensor characterization used a tube phantom (i.d.: 3/16 in., o.d.: 5/16, BD Intramedic polyethylene tubing PE#160 held in a 3D-printed housing). Tubes were filled with 40 μL of AuPD solution, with AuPD solution plus BSA (2 μg/mL, 5 μg/mL), or with AuPD solution plus caspase-3 (2 μg/mL, 5 μg/mL) incubated at 37 °C for 24 or 48 h. The US/PA images were acquired using an LZ250 transducer (256 elements, 18.5 MHz center frequency) with the Vevo LAZR system (FujiFilm Visualsonics, Inc.). For 3D imaging, the samples were imaged at 532, 700, 750, and 800 nm wavelengths over 10.21 mm elevational range with 0.152 mm steps. The final tube phantom images were reconstructed in the coronal view using maximum intensity projection in VevoLAB version 5.7.0 (FujiFilm Visualsonics, Inc.). For spectroscopic PA analysis, the phantom was imaged using wavelengths from 680 to 970 nm in 2 nm intervals. For both the quantitative PA analysis and the spectroscopic PA analysis, background subtraction was performed using water-only PA amplitude images at corresponding wavelengths and laser fluences, and the PA amplitudes were calculated using VevoLAB 5.7.0 software.

### Cell Culture

Human adipose-derived mesenchymal stem cells (Lonza Biosciences, Basel, Switzerland) were cultured in DMEM, supplemented with 10% FBS and 1% penicillin/streptomycin. Cells were incubated under standard conditions at 37 °C in a 5% CO_2_ humidified incubator (Heracell VIOS 160I, Fisher Scientific). Culture media was changed every 3 days, and cells were passaged at ∼80–90% confluency. hADMSCs were detached using 0.05% Trypsin-EDTA, neutralized with DMEM, centrifuged, counted with a hemocytometer, and finally seeded onto fresh cell culture T75 or T175 flasks at approximately 5000 cells/cm^2^. Only hADMSCs between passages P4 and P9 were used.

### Stem Cell Labeling with AuPD

To study cellular uptake of AuPD, hADMSCs were harvested, counted using a hemocytometer, and plated in a six-well plate (200,000 cells/well, 2 mL media volume). After 24 h, old cell media was aspirated, cells were washed with PBS (3 times), and media containing AuPEG (control) or AuPD (0.5 1.0, or 2.0 nM) was added. After incubation with nanoparticles for 24 h, the labeled cells were washed with PBS (3 times) to remove any residual nanoparticles and harvested for experimental studies. For all *in vitro* experiments, the AuPEG-labeled and AuPD-labeled hADMSCs were fixed in 4% PFA (Sigma-Aldrich) for 30 min and washed with PBS (3 times). Cells were then pelletized and analyzed for colorimetric studies. To assess cell uptake with bright-field microscopy (Zeiss AxioObserver), we fixed and stained the AuPEG-labeled and AuPD-labeled hADMSCs with eosin.

To evaluate the US/PA signal from AuPD-labeled hADMSCs, a tissue mimicking gelatin dome phantom was created. The phantom base consisted of 8% gelatin (MP Biomedicals) and 0.2% silica (Sigma-Aldrich) by mass. Dome-shaped inclusions were then prepared by mixing equal parts of AuPEG-labeled (0.5 1.0, and 2.0 nM) or AuPD-labeled (0.5 and 1.0 nM) hADMSCs with 16% gelatin solution to give a final gelatin concentration of 8%. All domes had a final cell concentration of 500 cells/μL. Each dome was imaged with US/PA imaging at 532, 700, 750, and 800 nm wavelengths. PA spectral imaging was conducted at 680–970 nm at 2 nm intervals. All data was processed using VevoLAB 5.7.0 software and analyzed in ImageJ (1.53n).

### *In Vitro* Toxicity Evaluation of AuPD

hADMSCs were seeded in a 96-well plate at an initial density of 10,000 cells/well and incubated at 37 °C under 5% CO_2_ for 24 h. Next, the cells were labeled with AuPD at concentrations of 0, 0.25, 0.50, 1.00, 2.00, and 4.00 nM. The 0 nM group acted as a control. The cells were incubated for another 24 h, after which they were washed thoroughly with PBS (3 times) to remove any residual nanoparticles. Then, 0.5 mg/mL of MTT in culture media was added to each well and incubated for 4 h. The solution was then aspirated, and 200 μL of DMSO was added to each well for complete dissolution of the formazan salt. The absorbance at 590 nm was measured using a multiwell plate reader (Synergy HT, BioTek) to evaluate cell viability. Similarly, the MTT assay was also performed after cell incubation with DOX at multiple concentrations (0, 5, 10, and 20 μM) to determine the DOX concentration that induced mild apoptosis. Cell survival, as determined by the MTT assay, was normalized to readings from control cells.

Cellular apoptosis due to AuPD and DOX was also evaluated by using an Annexin V-FITC apoptosis detection kit (Sigma-Aldrich). In brief, hADMSCs were cultured in a six-well plate at 100,000 cells/well for 24 h, exposed to AuPD (0.5 nM) and/or DOX (20 μM) for 24 h, washed with PBS (3 times), and harvested, followed by Annexin V-FTIC staining according to the protocol provided by the manufacturer. Cells were then fixed on slides and imaged by using a confocal microscope (Zeiss LSM 700). The images were analyzed using Zen Blue software (Zeiss Inc.).

### *In Vitro* Tridifferentiation Assay

To conduct the tridifferentiation assay, hADMSCs were first seeded onto a six-well tissue culture plate at suitable cell densities for the respective lineages, described in detail below. Cells with low passage numbers (<5) were used for the differentiation experiments. Cells in the experimental group were labeled with AuPD (0.5 nM, 24 h), while AuPD-negative (control) cells were not labeled with AuPD. Cells were incubated at 37 °C in a humidified atmosphere containing 5% CO_2_ throughout the process. After AuPD labeling and incubation, the cells were washed three times with PBS to remove any extracellular AuPD and the standard media was replaced with media to induce adipogenic, chondrogenic, or osteogenic differentiation. As a second control (“noninduced control”), both AuPD-labeled cells and AuPD-negative cells were grown in standard media (DMEM, supplemented with 10% FBS and 1% penicillin/streptomycin), i.e., media that does not trigger adipogenic, chondrogenic, or osteogenic differentiation.

Adipogenic differentiation was induced with the StemPro adipogenesis differentiation kit (Thermo Fisher Scientific) according to the manufacturer’s guidelines. MSCs were plated in six-well tissue culture plates at a concentration of 1 × 104 cells/cm^2^. Cells in some wells were labeled with AuPD, while other wells served as a control without AuPD addition. After labeling, standard media was replaced with complete adipogenesis differentiation medium, or cells were maintained in standard media (noninduced control). Media were changed for all cells every 2–3 days. After 14 days, cells were washed and stained with Oil Red O (Millipore Sigma, according to the manufacturer’s instructions) for the detection of neutral lipids, which indicates adipogenic differentiation.

Chondrogenic differentiation was induced with the StemPro chondrogenesis differentiation kit (Thermo Fisher Scientific) according to the manufacturer’s guidelines. Cell solutions of 1.5 × 10^7^ viable cells (both AuPD-labeled and without AuPD labeling) were generated and dropped at multiple locations on six-well tissue culture plates. After cultivating these micromass cultures for 2 h under high-humidity conditions, standard media was replaced with complete chondrogenesis differentiation medium, or cells were maintained in standard media (noninduced control). Media was changed for all cells every 2–3 days. After 16 days, cells were washed and stained with Alcian Blue (Millipore Sigma, according to the manufacturer’s instructions) for the detection of polysaccharides to indicate chondrogenic differentiation.

Osteogenic differentiation was carried out with the StemPro osteogenesis differentiation kit (Thermo Fisher Scientific) according to the manufacturer’s guidelines. Cells were plated in six-well tissue culture plates at a concentration of 5 × 10^3^ cells/cm^2^. After AuPD labeling, or without AuPD present for AuPD-negative controls, standard media was replaced with complete osteogenesis differentiation medium or refreshed with standard media (noninduced controls). Media were changed for all cells every 3–4 days. After 24 d, cells were washed and stained with Alizarin Red (Millipore Sigma, according to the manufacturer’s instructions) for the detection of calcium, which indicates osteogenic differentiation.

All stained cells were examined under BF microscopy (Zeiss AxioObserver), and the resulting images were analyzed using Zen Blue software (Zeiss).

### *In Vitro* Surface Marker Assay

hADMSCs with low passage numbers (<5) were seeded into a six-well tissue culture plate at 50,000 cells/well. Cells in the experimental group were labeled with AuPD (0.5 nM, 24 h), while negative control cells were unlabeled. After AuPD labeling, cells were washed three times with PBS to remove any extracellular AuPD and the media was replaced with 4% PFA (pH 7.4) for 20–30 min to fix the cells. Cells were then washed three times with PBS and then exposed to anti-human CD14 (130-113-708, Miltenyi Biotec) or CD90 (130-117-684, Miltenyi Biotec) antibodies (1:50 dilution in PBS). The cells were left at room temperature on a shaker plate for 10 min, washed three times with PBS to remove unbound antibodies, and visualized by confocal fluorescence microscopy (Zeiss 700B). The resulting images were analyzed using Zen Black software (Zeiss).

### *In Vitro* Sensor Activity

The levels of caspase-3 due to AuPD-labeling or DOX treatment were first assessed using a human active caspase-3 ELISA kit according to the protocol provided by the manufacturer (Invitrogen). To also evaluate stem cell apoptosis, AuPD aggregation within hADMSCs was monitored. In brief, hADMSCs were cultured in six-well plates, labeled with AuPD (0.5 nM), and treated with DOX (20 μM) for 48 h to induce apoptosis. Unlabeled hADMSCs and AuPD-loaded cells without DOX were used as controls. AuPD aggregation within labeled hADMSCs under apoptotic stress was examined by using both bright-field microscopy (Zeiss AxioObserver) and dark-field microscopy (Leica DM4000). To monitor the change in PA signal due to apoptosis, a tissue mimicking 8% gelatin phantom was created as described above. Inclusions were prepared by mixing equal parts of the unlabeled hADMSCs, or AuPD-labeled hADMSCs with and without DOX, with 16% gelatin solution. All domes had a final concentration of 500 cells/μL. US/PA imaging was performed using an LZ250 transducer at 532, 700, 750, and 800 nm wavelengths. Each dome was imaged with PA spectral scanning from 680 to 970 nm at 2 nm intervals. All the data were processed using VevoLAB 5.7.0 software and analyzed in ImageJ software (1.53n).

### Hindlimb Ischemia Model

All animal procedures were approved by the Institutional Animal Care and Use Committee (IACUC) at the Georgia Institute of Technology in accordance with federal guidelines for the care and use of laboratory animals. We used a unilateral hindlimb ischemia model following well-established methods.^56,57^ In brief, all studies used 6–8-week-old (*n* = 3 control and *n* = 6 experimental) female Nu/Nu mice (Charles River). Aseptic surgical techniques were followed. Anesthesia was induced with 5% isoflurane and 0.4–0.8 L/min oxygen in an induction chamber. Once immobilized, the mice were moved to a heating pad on a sterile surgical table and placed in a supine position. The surgical site was cleaned and swabbed with alcohol wipes and Betadine three times. An incision was made in the skin. The skin was retracted to expose the underlying neurovascular bundle. The femoral artery and vein were carefully separated from the nerve and were then ligated immediately proximal to the femoral profunda and ligated again ∼1 cm distal to the femoral profunda using 5–0 sutures. This ligated region was excised by carefully cutting between the sutures and removing the vessels. The skin incision was closed with wound clips and surgical glue. A single dose of sustained release buprenorphine (0.08 mg/kg intraperitoneal) was administered. The animal was allowed to recover on a heating pad prior to returning to housing. Animals were monitored following surgery, and additional pain relief was administered for 3 days following the procedure (ketoprofen; 5 mg/kg subcutaneous).

### *In Vivo* Sensor Activity

AuPD-labeled hADMSCs (0.5 nM AuPD, 24 h incubation, 500,000 cells/25 μL) were washed to remove any excess AuPD, harvested, counted with a hemocytometer, and resuspended in phenol red-free DMEM (Cytiva). Then, they were mixed with an equal volume of Matrigel (25 μL, Corning) to create a final injection solution of 50 μL containing AuPD-labeled hADMSCs. Unlabeled hADMSCs in Matrigel and Matrigel-only injections were used as controls at similar volumes and cell concentrations. All injections and solutions were prepared on ice and injected into the outer hindlimb area in mice. US/PA imaging was performed longitudinally at days 0 (immediately after injection), 3, 5, 7, 10, 12, and 14.

For imaging studies, the mice were anesthetized using inhaled isoflurane (2–3%) mixed with 100% oxygen (0.4–0.8 L/min) and placed on a heated, motorized translational stage with an anesthesia cone. Per IACUC guidelines, animals were never imaged on consecutive days and each imaging session was completed within 4 h. All *in vivo* US/PA images were acquired using the LZ550 transducer coupled with a fiber optic cable (256 elements, 40 MHz center frequency) for better anatomical resolution. PA spectral analysis cine loops from 680 to 970 nm were acquired at three different slices overlying the injection site, the two extreme edge regions, and the center region, with the center slice of the injected region being displayed as the representative slice in Figures 5c and 6a. Images were acquired from 680 to 970 nm at 2 nm wavelength intervals. The two edge slices were used as boundaries to perform further US/PA 3D imaging, with the transducer attached to a translational motorized stage to acquire 2D cross-sectional images at wavelengths of 532, 700, 750, and 800 nm at 0.152 mm intervals. All data was exported, preprocessed, and analyzed using VevoLAB5.7.0 software and ImageJ (1.53n).

To construct slope analysis images, the PA spectral scans were loaded in Matlab (R2020a) and compiled into a 3D spectral matrix with each 2D slice containing PA amplitudes from 700 to 900 nm at 5 nm intervals. Then, slopes were computed pixelwise by linear least-squares regression of the PA amplitude on wavelength and used to construct a “slope image”. This image was then filtered using a 2D Gaussian filter with a smoothing kernel having a standard deviation of 2.0, thresholded to remove noise, and finally overlaid onto the corresponding US image.

### Statistics

For *in vitro* experiments, at least three technical replicates were run for each set of experimental conditions. For *in vivo* experiments, at least three independent biological replicates (animals) per group were used for control conditions as follows: (i) Matrigel-only injection without ischemia-inducing surgery, (ii) Matrigel injection with unlabeled hADMSCs without ischemia-inducing surgery, and (iii) Matrigel-only injection with ischemia-inducing surgery. Six independent biological replicates per group were used for the experimental conditions: (iv) AuPD-labeled hADMSC injection without ischemia-inducing surgery and (v) AuPD-labeled hADMSC injection with ischemia-inducing surgery. Different synthesis batches of AuPD nanosensors and stem cells were used in a randomized pattern to create the AuPD-labeled stem cells. All replicates used for a single experiment were technical replicates since the hADMSCs were sourced from a single donor; however the donors were different between experiments. For experiments involving two groups, control and experimental samples were compared by an unpaired Student’s *t* test since each sample replicate was independent of every other sample replicate, and the replicate data arose from a randomized process. A Jarque-Bera goodness-of-fit test was conducted on all data to confirm that the skewness and kurtosis matched a normal distribution prior to the Student’s *t* test. For experiments involving three or more groups, experimental conditions were compared using one-way or two-way ANOVA with replicated measurements followed by a *post hoc* Dunnett test to calculate the effective size and the confidence interval limits (*p < 0.05, **p < 0.01, ***p < 0.001) as appropriate. Unless otherwise noted, all statistical analyses were performed using Microsoft Excel, R, or GraphPad Prism version 8.0.1 (GraphPad Software, La Jolla, CA, USA).

## Data Availability

The authors confirm that the data supporting the findings of this study are available within the article and its Supporting Information. Any additional data are available from the corresponding authors upon request.

## Supporting Information Available

In-depth characterization of the AuPD nanosensor in response to caspase-3; AuPD photostability; in-depth characterization of cellular uptake of AuPD *in vitro*; Annexin V assay for AuPD toxicity; Tridifferentiation assay for AuPD-labeled MSC functional assessment; Surface marker analysis for AuPD AuPD-labeled functional assessment; BF images of AuPD aggregation; DF images of AuPD controls; in-depth characterization of sensor activity of AuPD *in vitro*; US/PA imaging of additional negative controls; 14-day longitudinal imaging of cell tracking and apoptosis; 3D cross-sectional and maximum intensity projection US/PA images of cell tracking and apoptosis; postimaging histology analysis (PDF)
Spectral and 3D US/PA imaging video (MP4)
Spectral and 3D US/PA imaging video (MP4)
