# Supplementary material for: In Vivo Photoacoustic Monitoring of Stem Cell Location and Apoptosis with Caspase-3-Responsive Nanosensors
Source: ACS Nano. 2023 Sep 13;17(18):17931–45. doi: 10.1021/acsnano.3c04161 (PMC10540261; doi:10.1021/acsnano.3c04161)
Supplement: Supplementary file 1 — nn3c04161_si_001.pdf [file nn3c04161_si_001.pdf]

Supporting Information

In vivo photoacoustic monitoring of stem cell location and apoptosis with caspase-3-responsive nanosensors

Anamik Jhunjhunwala[1,†], Jinhwan Kim[1,2,†], Kelsey P. Kubelick[1,2], C. Ross Ethier[1,*], and Stanislav Y. Emelianov[1,2,*]

[1] *Wallace H. Coulter Department of Biomedical Engineering, Georgia Institute of Technology and Emory University School of Medicine, Atlanta, GA 30332, USA*

[2] *School of Electrical & Computer Engineering, Georgia Institute of Technology, Atlanta, GA 30332, USA*

[†] These authors contributed equally to this work

[*] Corresponding authors: C. Ross Ethier and Stanislav Y. Emelianov

✉E-mail: ross.ethier@bme.gatech.edu and stas@gatech.edu

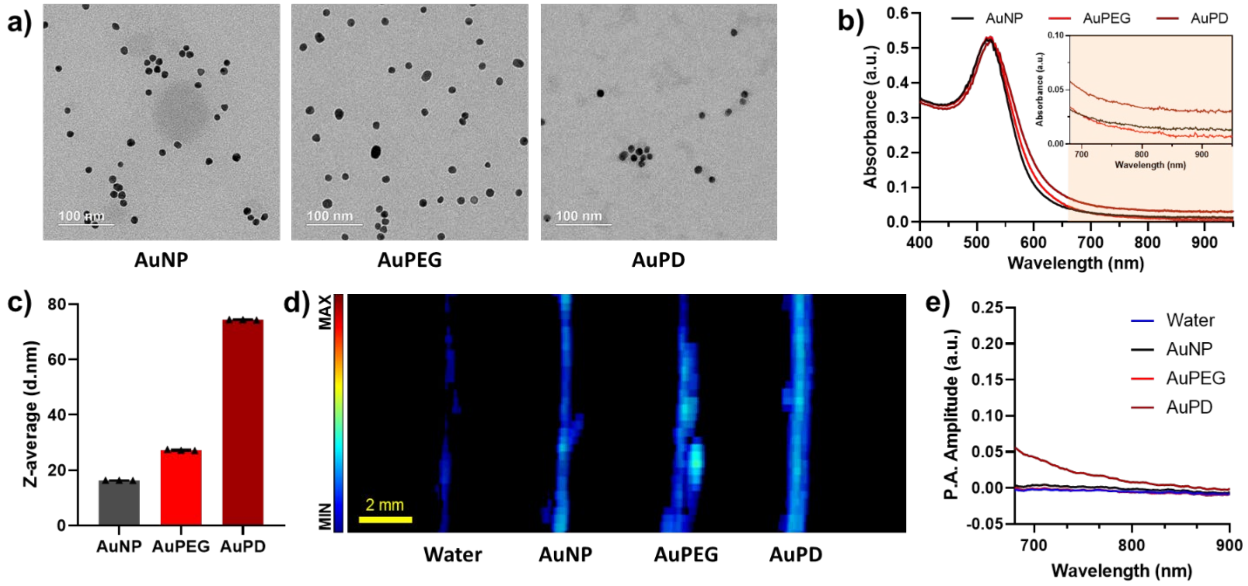

Figure S1. Characterization of AuPD nanoparticles and comparison with AuNP and AuPEG precursors. (a) Transmission electron micrographs of AuNP, AuPEG, and AuPD. Scale bar = 100 nm. No observable change in the morphology of the AuNPs is evident after surface functionalization by PEG and the triblock peptide. Some mild aggregation is noted after functionalization by the triblock peptide which allows for tracking of non-apoptotic AuPD-labeled stem cells. (b) UV-vis spectra of AuNP, AuPEG, and AuPD. The shaded region represents the NIR-I optical window. Inset shows magnified region (680 – 900 nm). (c) Hydrodynamic diameters of AuNP, AuPEG, and AuPD. There is a slight increase in size of AuPEG due to surface functionalization with PEG and of AuPD due to mild aggregation upon functionalization with the triblock peptide. (d) PA amplitude of water, AuNP, AuPEG, and AuPD in tube phantoms. (e) PA spectra of water, AuNP, AuPEG, and AuPD from 680-900 nm (n=3; solid line is mean). (d) and (e) show the potential of AuPD for longitudinal tracking of non-apoptotic AuPD labeled stem cells.

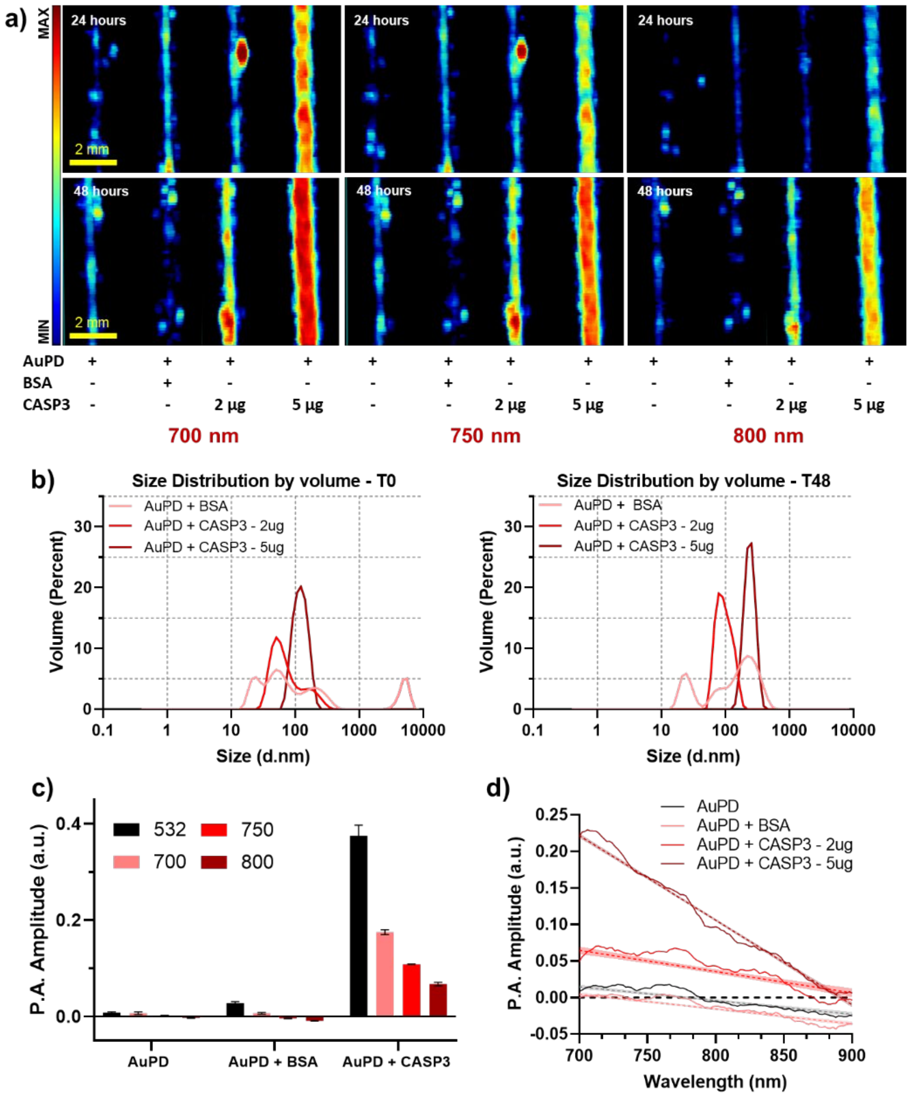

Figure S2. Characterization of the caspase-3-responsive behavior of AuPD. (a) PA images of tube phantoms containing AuPD only (left), AuPD with BSA (middle left), AuPD with caspase-3 (2µg/ml, middle right), and AuPD with caspase-3 (5µg/ml, right) after 24 h (top) and 48 h (bottom) of incubation (wavelength = 700nm – left column, 750nm – middle column, and 800 nm – right column). Aggregation of AuPD in the presence of caspase-3 results in a significant increase in the PA signal. (b) Volume-weighted hydrodynamic size distributions of AuPDs nanoparticles incubated with BSA (control) and caspase-3 (2µg/ml, 5µg/ml) at 0 h and 48 h, showing increased aggregation upon incubation with caspase-3, with only modest aggregation in the presence of control BSA. Plotted data are average of n=3 technical replicates. T0 and T48 refer to measurements made at the time of initiation of incubation, and 48 hours after incubation, respectively. (c) Quantitative PA amplitude of AuPD incubated for 24 h with BSA or caspase-3 (5µg/ml) measured in the tube phantom (wavelengths = 532nm, 700nm, 750nm, and 800 nm) (n=3). (d) Spectroscopic PA amplitude of AuPD with BSA and caspase-3 (2µg/ml, 5µg/ml) at 24 h from 700 – 900 nm. Dashed line represents PA = 0.00 a.u. Dotted lines represent best fit linear regressions from 700 – 900 nm. Shaded regions represent 95% CI of the linear regressions. (n=3). Note the change in slope of AuPD PA spectra upon incubation with caspase-3.

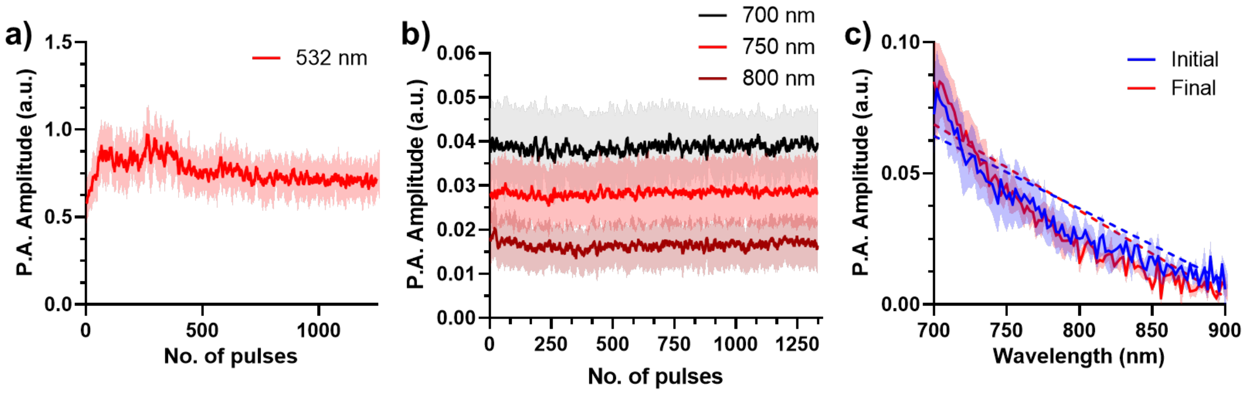

Figure S3. Photostability of AuPD. (a) PA amplitude of AuPD under 532 nm laser irradiation for 5 min (n=3; solid line is mean, while shaded regions represent the standard deviation). (b) PA amplitude of AuPD under 700, 750, and 800 nm laser irradiation for 5 min each (n=3; solid line is mean, while shaded regions represent the standard deviation). (a) and (b) show no difference in photostability under >1250 laser pulses at different wavelengths. (c) Spectroscopic PA amplitude of AuPD before vs. after all laser irradiation over 20 mins, consisting of 5 mins each at 532, 700, 750 and 800 nm (n=3; solid line is mean, while shaded regions represent the standard deviation). Dashed lines represent best fit linear regressions from 700 – 900 nm. There is no observable difference in the PA spectra after 20 mins of laser irradiation at different wavelengths, showing the photostability of AuPD.

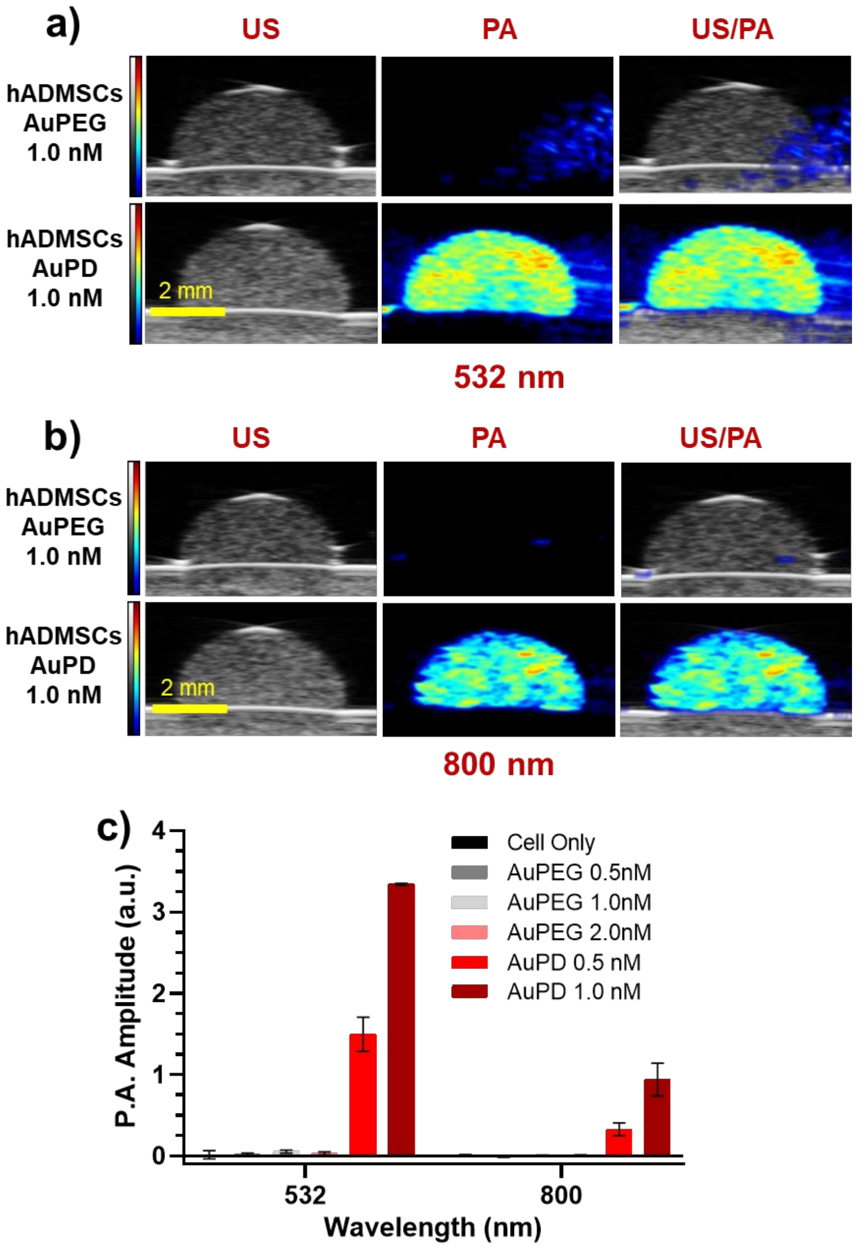

Figure S4. Characterization of the cellular uptake of AuPD. (a-b) US (left column), PA (middle column) and US/PA (right column) images of a gelatin dome phantom containing AuPEG-labeled hADMSCs (1.0 nM, top row) and AuPD-labeled hADMSCs (1.0 nM, bottom row) imaged at (a) 532 nm and (b) 800 nm wavelengths. (c) Quantitative PA amplitudes of hADMSCs only, AuPEG-labeled hADMSCs (0.5nM, 1.0nM, 2.0nM), and AuPD-labeled hADMSCs (0.5nM, 1.0nM) at 532 nm and 800 nm from the dome phantom (n=3). The PA amplitude is averaged over the entire dome phantom and averaged over three independent domes. The mean of the average PA amplitude of the three domes is plotted, with error bars representing standard deviations.

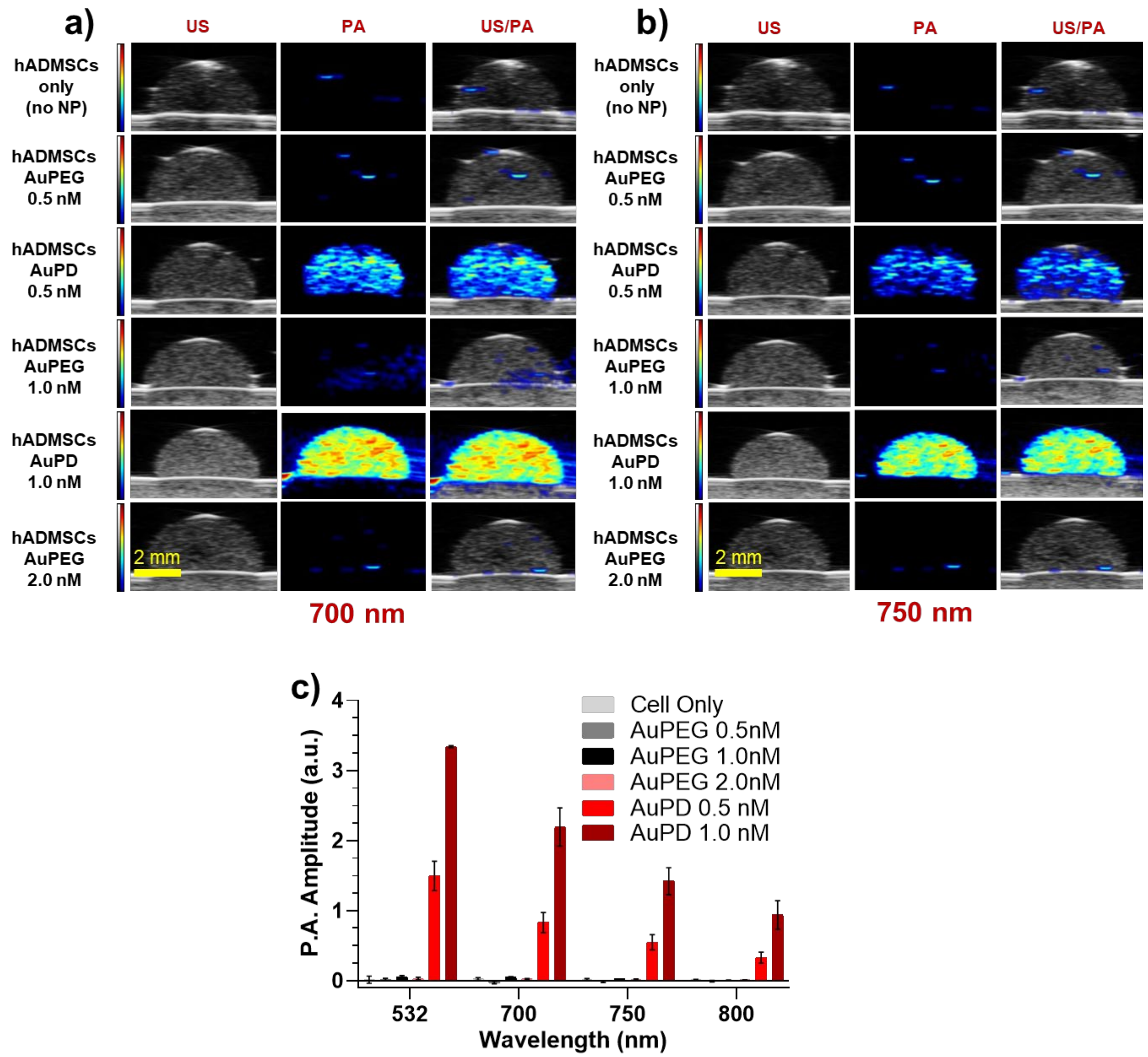

Figure S5. Characterization of the cellular uptake of AuPD. (a-b) US (left column), PA (middle column) and US/PA (right column) images of a gelatin dome phantom containing hADMSCs only (top row); hADMSCs labeled with AuPEG at 0.5nM (second row), 1.0nM (fourth row), or 2.0nM (sixth row); and hADMSCs labeled with AuPD nanoprobes at 0.5nM (third row) or 1.0nM (fifth row) imaged at (a) 700 nm and (b) 750 nm wavelengths. (c) Quantitative PA amplitudes at 532, 700, 750 and 800 nm from the dome phantom (n=3). Note the significantly greater cellular uptake of the AuPD compared to the AuPEG control at the same concentration due to the triblock peptide. Since the signal from 1.0 nM AuPD was very high, we chose 0.5 nM as the labeling concentration going forward. The PA amplitude is averaged over the entire dome phantom and averaged over three independent domes. The mean of the average PA amplitude of the three domes is plotted, with error bars representing standard deviations.

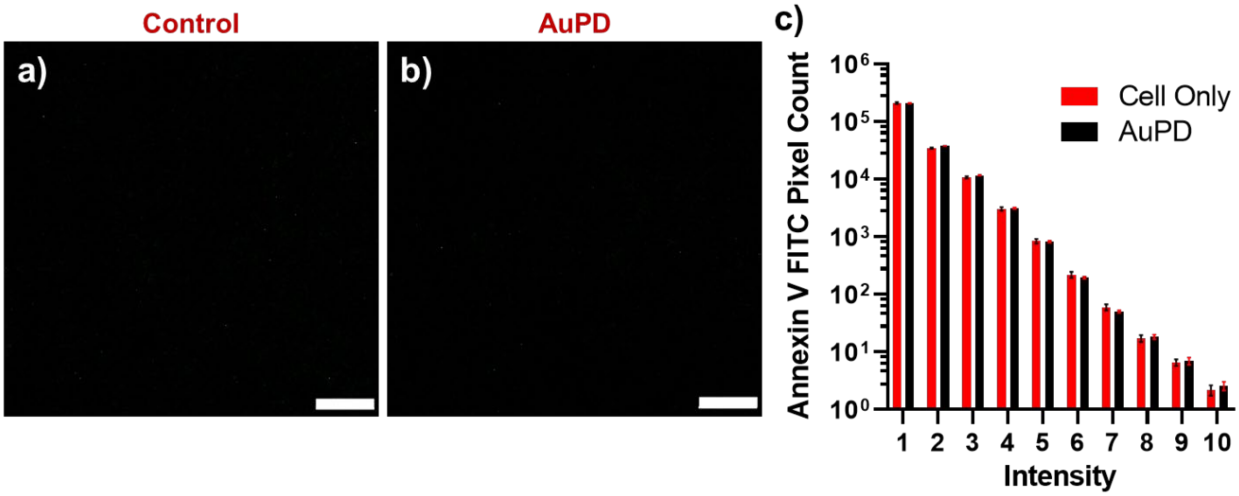

Figure S6. Characterization of the *in vitro* toxicity of AuPD using Annexin V-FTIC staining. (a-b) Confocal fluorescence microscopy image of (a) control hADMSCs and (b) hADMSCs + AuPDs stained with FITC-tagged Annexin V. Scale bar = 200 µm. In (a) and (b), minimal fluorescence is observed, indicative of minimal cell death confirming minimal cytotoxicity due to AuPD labeling. (c) Histogram of Annexin V-associated fluorescence intensity from hADMSCs and hADMSCs + AuPD. Intensity represents channel brightness divided into ten equal bins, while the pixel count represents the number of pixels which fell in that intensity bin. We see no significant difference between the bin frequencies of the control vs. AuPD labeled hADMSCs, indicating negligible effect of AuPD on cell viability. A positive control for Annexin V labelling is shown in Supplementary Figure 9.

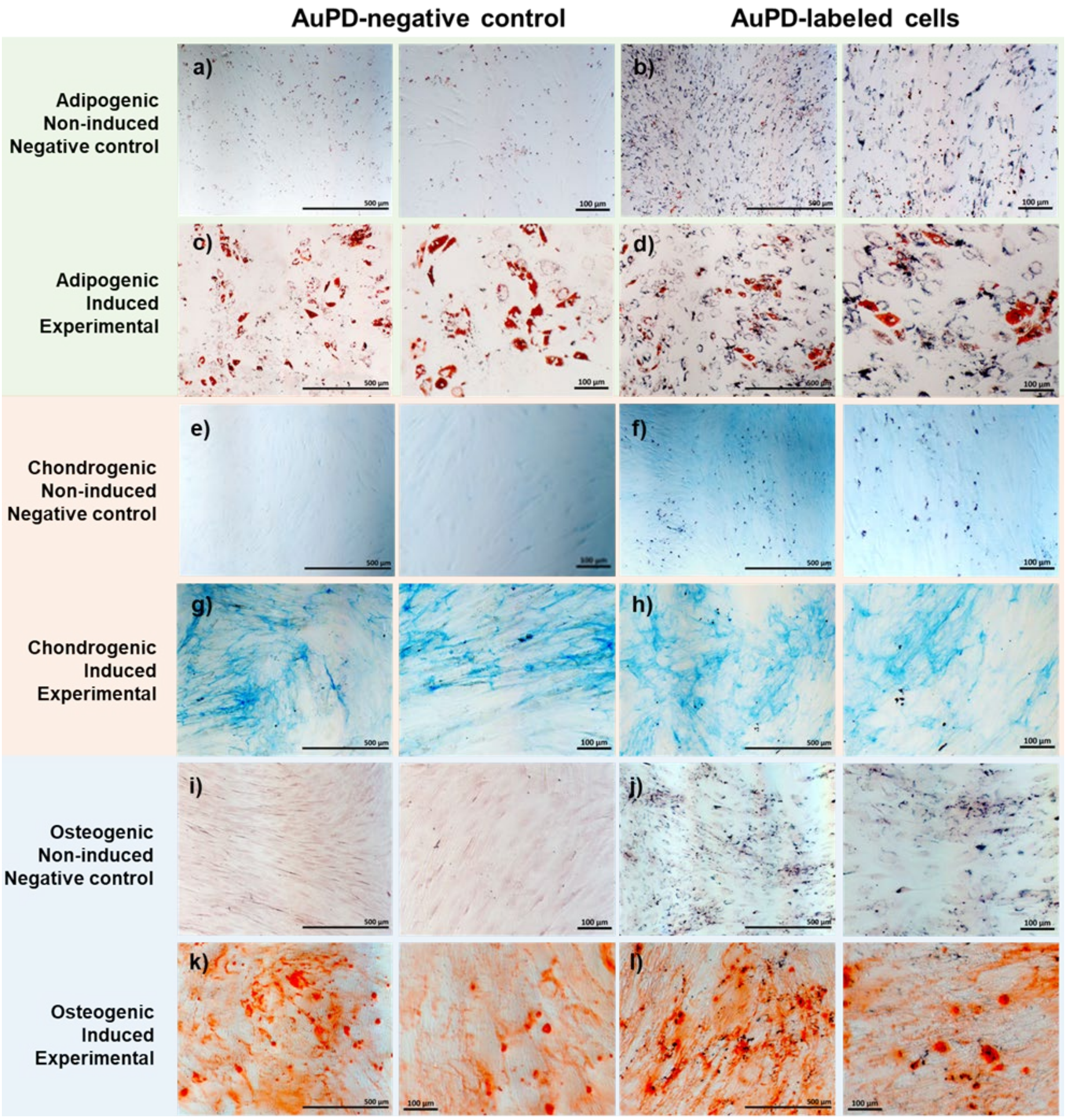

Figure S7. Tridifferentiation assay. Histological images confirm that the adipogenic, chondrogenic, and osteogenic differentiation capacity of MSCs remained unchanged by the presence of AuPDs. For each differentiation lineage – adipogenic (rows 1 and 2), chondrogenic (rows 3 and 4) and osteogenic (rows 5 and 6) – the images in the top row (rows 1, 3, and 5) show noninduced control hADMSCs (cells cultured in standard growth media), while the bottom row shows hADMSCs cultured in either adipogenic (row 2), chondrogenic (row 4), or osteogenic (row 6) differentiation media. The images in columns 1 and 2 show unlabeled (AuPD-negative) control cells while the images in columns 3 and 4 show AuPD-labeled cells. Columns 1 and 3 show large field of view images (scale bars:500 µm), while columns 2 and 4 represent magnified images (scale bars: 100 µm). We see no difference in differentiation capacity between AuPD-labeled and AuPD-negative (unlabeled) cells (n=3 technical replicates, representative images shown). Importantly, the presence of AuPD nanosensors did not induce any unintended differentiation, as confirmed by negative control conditions (cells cultured in non-inducing media), showing negative staining for the presence of differentiation markers.

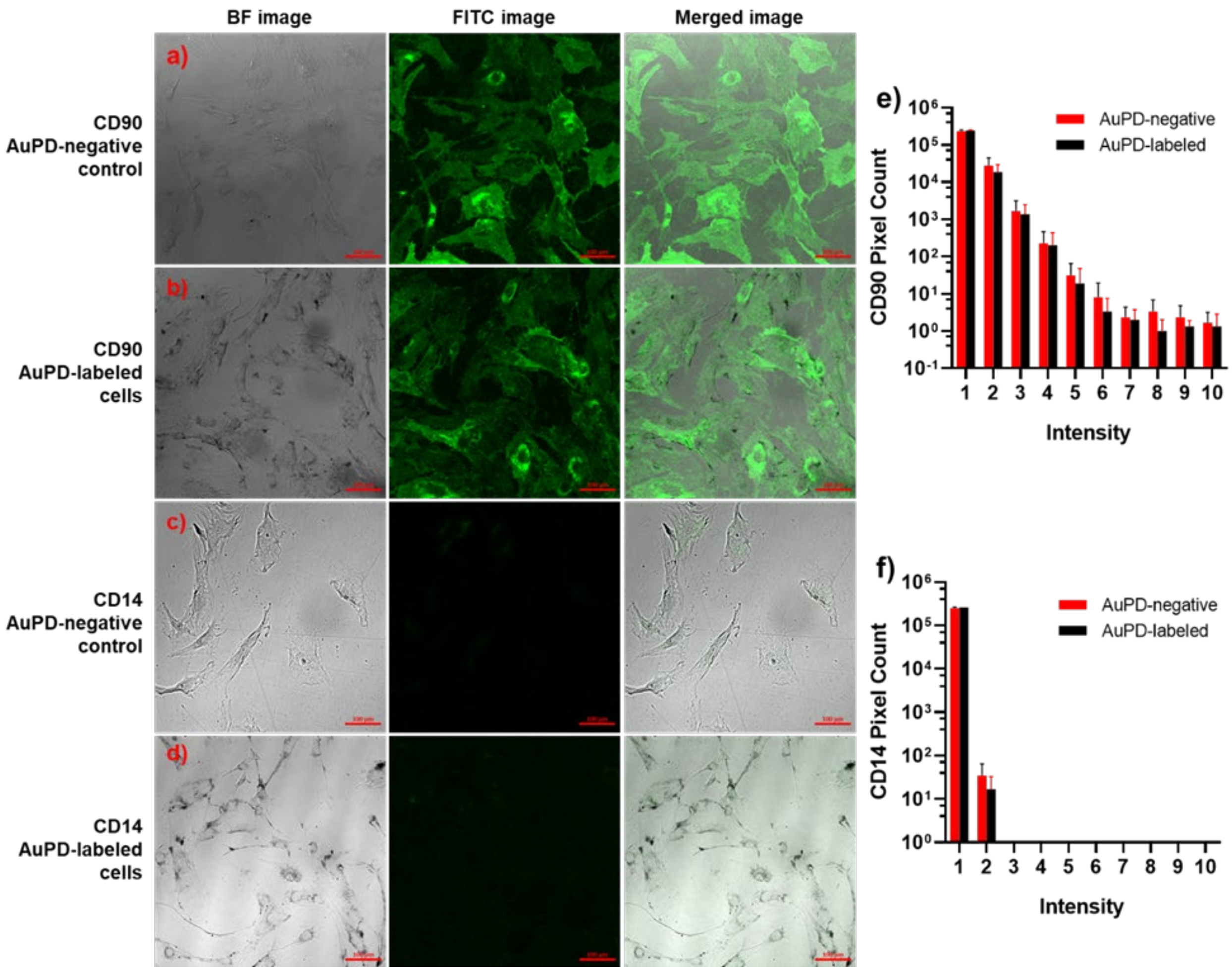

Figure S8. Cell surface marker labeling of AuPD-labeled hADMSCs. (a-b) Confocal fluorescence microscopy images of (a) control (AuPD-negative) hADMSCs and (b) AuPD-labeled hADMSCs stained with FITC-tagged CD90 anti-human antibody. The left column shows bright field (BF) images, the center column shows the FITC channel images, while the right column shows the merged images. Scale bar = 100 µm. In (a) and (b), significant FITC fluorescence is observed, indicative of the presence of the CD90 on the cell surface. Qualitatively there is no detectable difference between AuPD-negative (control) vs. AuPD-labeled cell populations. (c-d) Confocal fluorescence microscopy images of (c) control (AuPD-negative) hADMSCs and (d) AuPD-labeled hADMSCs stained with FITC-tagged CD14 anti-human antibody. The columns and scale bars are the same as panels (a) and (b). In (c) and (d), no FITC fluorescence is observed, indicative of the absence of CD14 on the cell surface. Qualitatively there is no evident difference between AuPD-negative (control) vs. AuPD-labeled cell populations. (e-f) Histograms of CD90 antibody-associated fluorescence intensity (e) and of CD14 antibody-associated fluorescence intensity (f) from AuPD-negative (control) and AuPD-labeled hADMSCs. Intensity represents channel brightness divided into ten equal bins, while the pixel count represents the number of pixels which fell in that intensity bin. We see no significant difference between the bin frequencies of the AuPD-negative (control) vs. AuPD-labeled hADMSCs in either panels (e) or (f), indicating negligible effect of AuPD labeling on cell surface CD14 and CD90 levels.

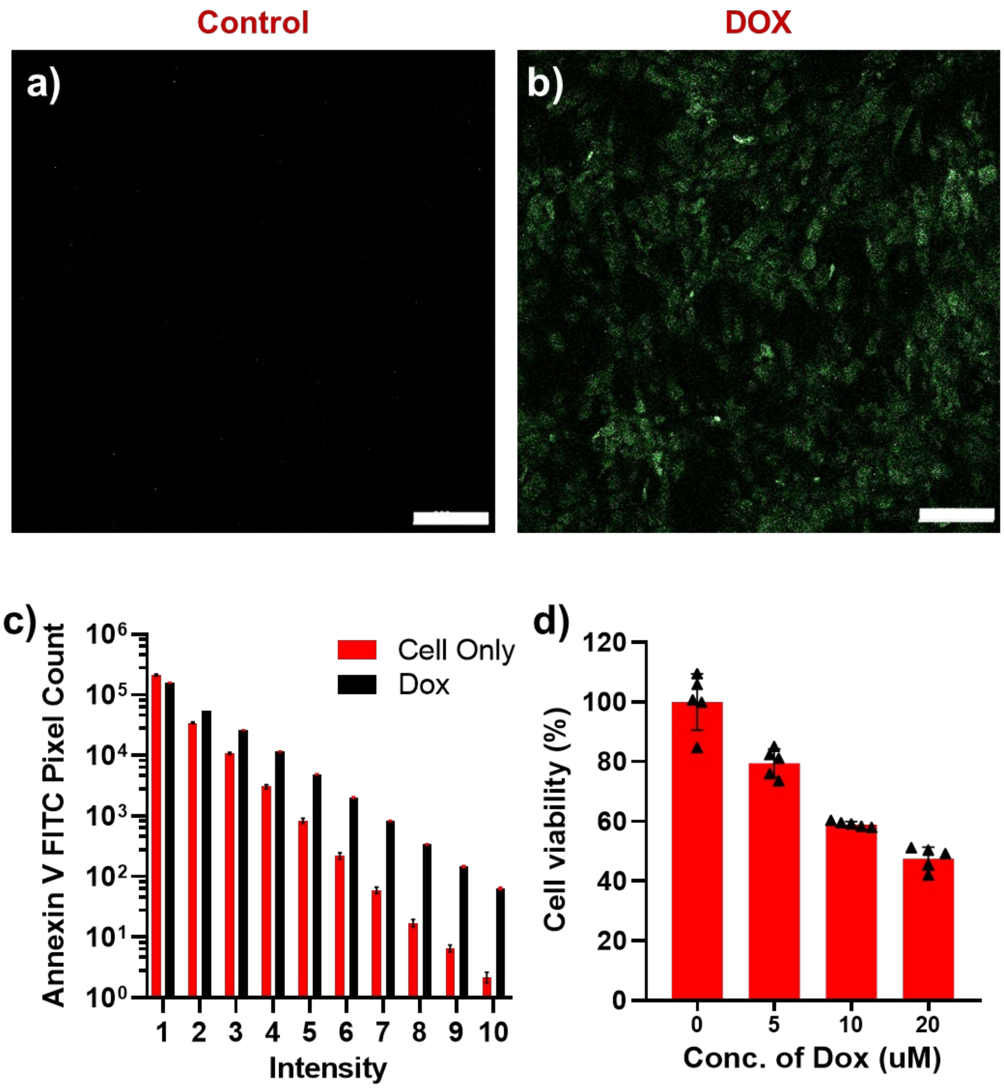

Figure S9. Characterization of the *in vitro* toxicity of DOX using Annexin V-FTIC staining. (a-b) Confocal fluorescence microscopy image of (a) control hADMSCs and (b) hADMSCs + DOX stained with FITC-tagged Annexin V. Scale bar = 200 µm. Note the high FITC expression in the DOX-incubated cells indicating apoptosis. (c) Histogram of fluorescence intensity from control hADMSCs and hADMSCs + DOX, as described in Supplemental Figure 6c. We see significantly greater bin pixel counts for the higher intensity bins for the DOX-incubated hADMSCs vs. control hADMSCs indicating higher FITC expression associated with apoptosis. (d) Cell viability data of hADMSCs + DOX (0µM, 5µM, 10µM, 20µM; incubated for 24 hours) using the standard MTT cell viability assay shows ~50% cell death upon incubation with DOX at 20µM concentration (n=5 technical replicates).

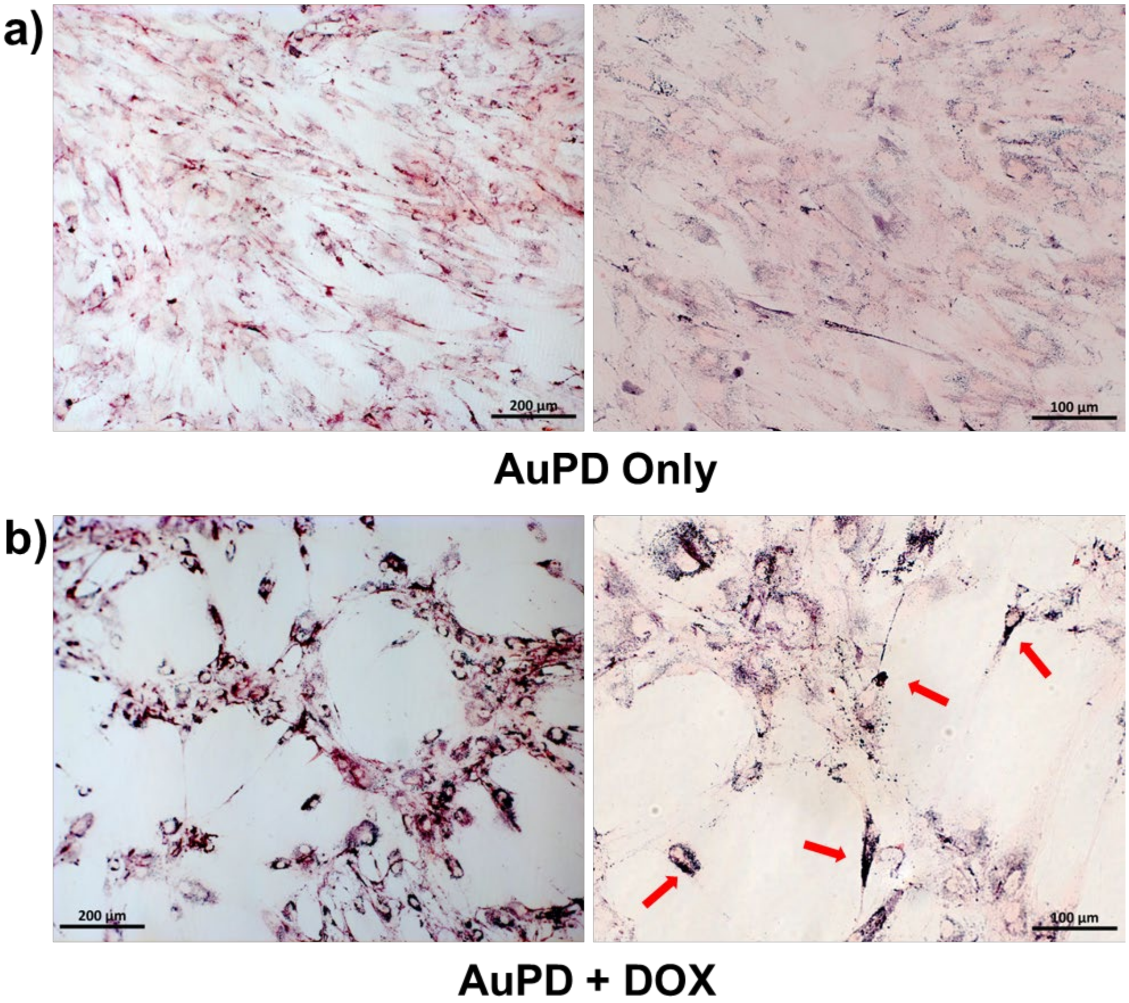

Figure S10. Bright-field images of (a) AuPD-labeled hADMSCs without DOX, and (b) AuPD-labeled hADMSCs with DOX (20 µM) incubated for 24 hours (scale bars = 200 µm, left and 100 µm, right). Aggregation of AuPD inside cells is indicated by red arrows. There is much greater aggregation of AuPD nanosensors in the DOX-incubated stem cells due to the presence of caspase-3.

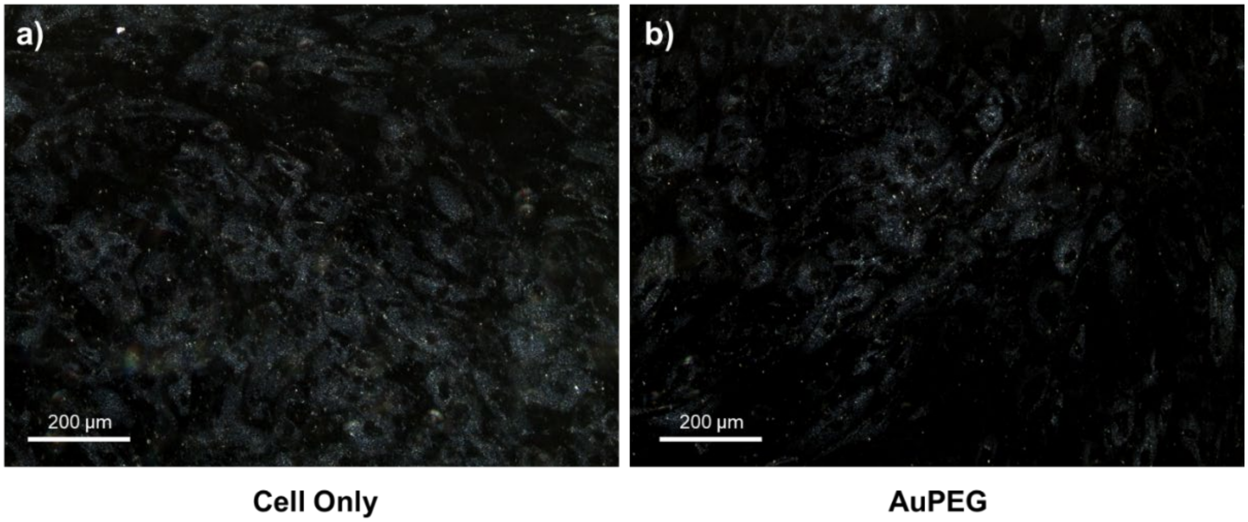

Figure S11. Dark-field images of (a) control hADMSCs and (b) AuPEG-labeled hADMSCs. Minimal scattering in both the cell-only control and AuPEG-labeled hADMSCs indicates the absence of nanoparticles and shows negligible cellular uptake of AuPEG by hADMSCs.

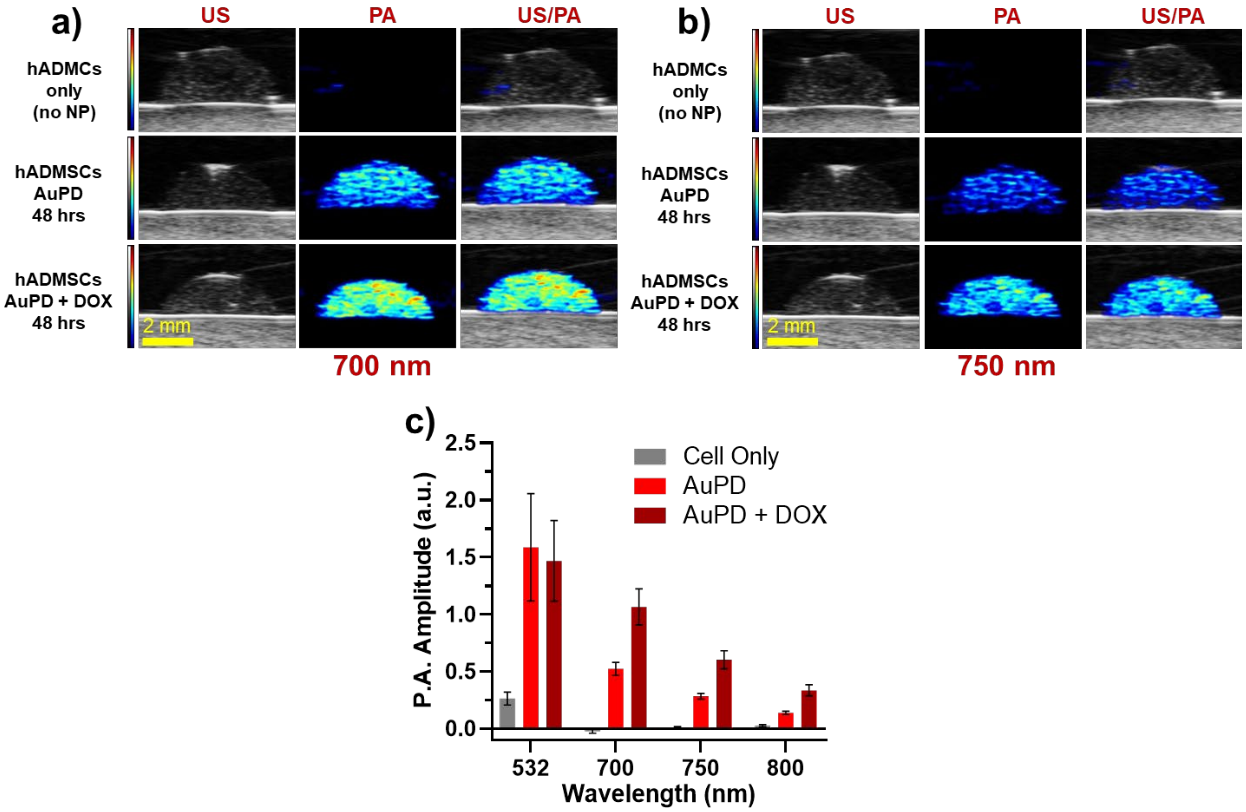

Figure S12. Characterization of the activity of the AuPD nanosensor *in vitro*. (a-b) US (left column), PA (middle column), and US/PA (right column) images of a gelatin dome phantom containing hADMSCs only (top row), AuPD-labeled hADMSCs without DOX (middle row), and AuPD labeled hADMSCs with DOX (20 µM) incubated for 48 h (bottom row) imaged at (a) 700 nm and (b) 750 nm wavelengths. (c) Quantitation of PA amplitude in gelatin phantoms containing control hADMSCs, and AuPD-labeled hADMSCs with and without DOX at 532 nm, 700 nm, 750 nm, and 800 nm (48 h, n=3). The PA amplitude is averaged over the entire dome phantom and averaged over three independent and randomly distributed domes. The mean of the average PA amplitude of the three domes is plotted, with error bars representing standard deviations.

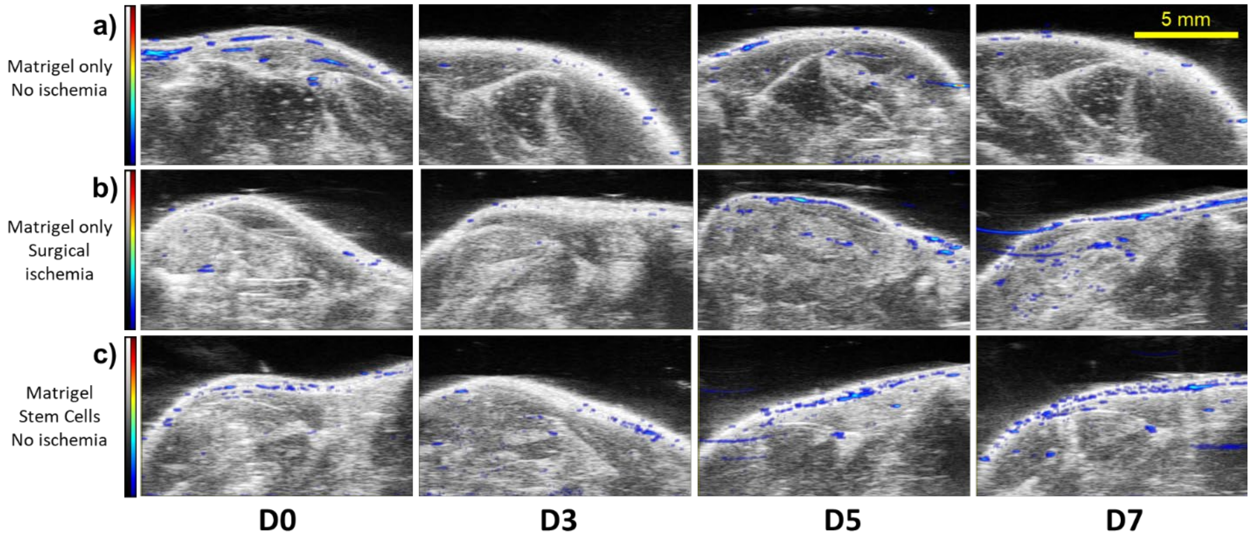

Figure S13. Characterization of the activity of the AuPD nanosensor *in vivo*. (a) Representative US/PA images (800 nm) from a mouse receiving intramuscular injection of Matrigel in a non-ischemic hindlimb at days 0, 3, 5 and 7. (b) As in (a), except that the injection occurred in a surgically-induced ischemic hindlimb. There is no observable PA signal from injection of only Matrigel, however US shows location of the injected matrigel. (c) Representative US/PA images (800 nm) from a mouse receiving intramuscular injection of unlabeled hADMSCs in Matrigel in a non-ischemic mouse hindlimb imaged at days 0, 3, 5 and 7. There is no observable PA signal from unlabeled stem cells or matrigel, and the location of injected unlabeled hADMSCs in Matrigel is also not apparent from the US image. In (a) the boundaries of the Matrigel-only injection are observable using US due to the differences in acoustic impedance between Matrigel and the surrounding tissue. However, in both (b) and (c) the boundaries become difficult to demarcate, due to surgical damage and unlabeled stem cells reducing the differences in acoustic impedance vs. surrounding tissue, respectively.

Supplementary Note 1

Motivated by reports of substantial apoptosis in delivered stem cells even in healthy mice, the non-ischemic model was observed for an additional week until day 14.[1–4] Conversely, observation of the surgical ischemia model was stopped on day 7 due to severe muscular atrophy in the mice. When observed until day 14, the PA signal in the non-ischemic mice increased with time and became comparable to the day 7 signal observed in the surgical ischemic mice, strongly suggesting rising levels of stem cell apoptosis even in a healthy mouse hindlimb (Figure S12a). Using spectroscopic PA analysis, we observed significantly greater PA amplitude in the surgical ischemia mouse model vs. the no ischemia mouse model until day 7, indicating rapid stem cell death due to an inhospitable hypoxic environment. This observation is consistent with our hypothesis that we could observe more apoptosis in the ischemic model vs. non-ischemic. However, this was followed by similar PA signal amplitudes in the non-ischemic model at day 14 vs. the surgical ischemia model at day 7, indicative of ongoing apoptosis even under control conditions (Figure S12b, c, d). Quantitation of PA amplitude at 800 nm showed that increase in signal for the non-ischemic model was significant at day 14 (5.9-fold higher vs. day 0) and was comparable to the signal from the surgical ischemia model at day 7 (Figure S12e). Slopes obtained from linear regression of PA spectra over the range 700-900 nm displayed similar trends, i.e., a constant increase in slope in the non-ischemic model (3.2-fold higher at day 14 vs. day 0; Figure S13b).

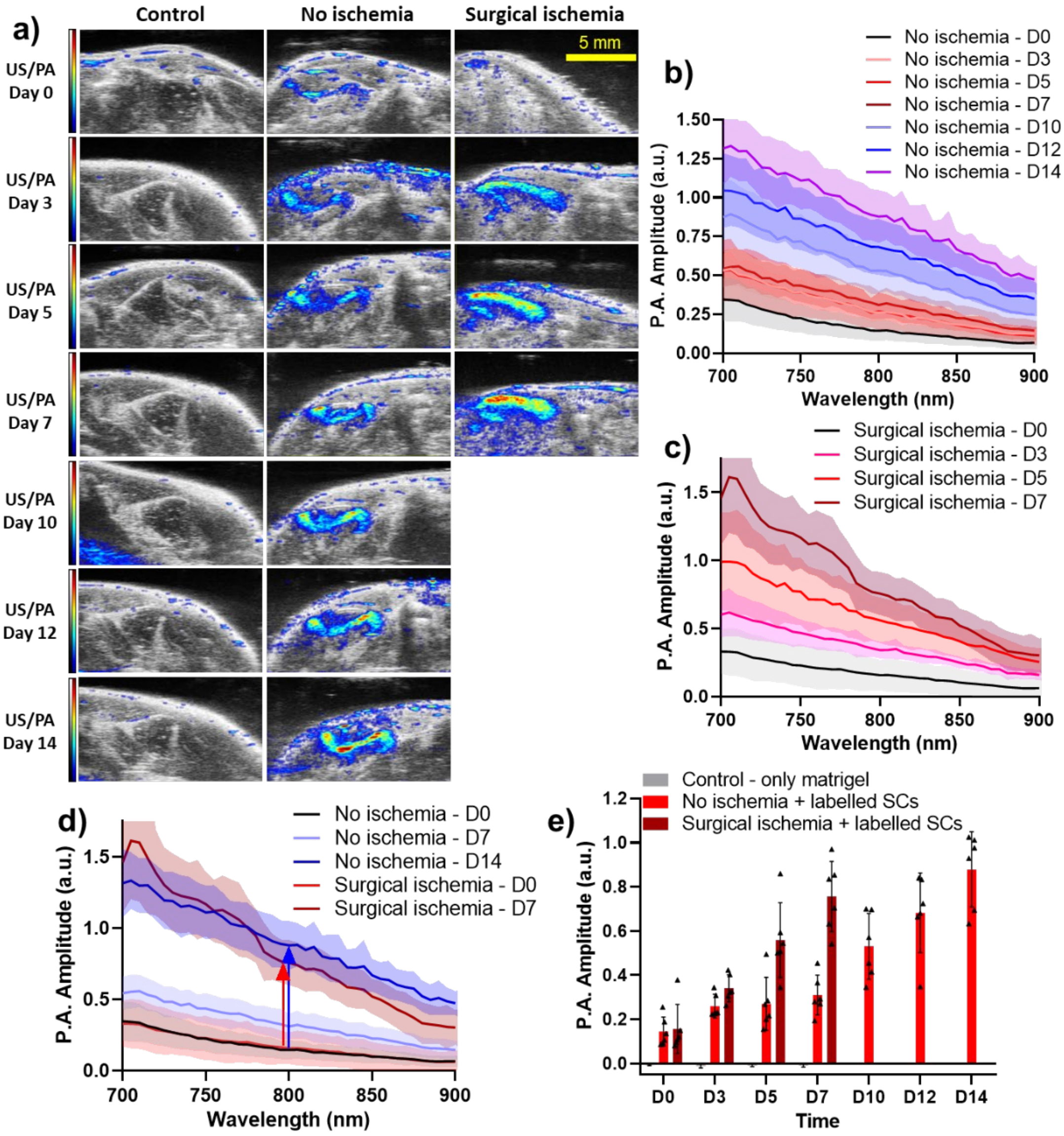

Figure S14. Characterization of the activity of the AuPD nanosensor *in vivo*. (a) Representative US/PA images (800 nm) of mice injected intramuscularly with: Matrigel-only control in a non-ischemic mouse (left column), AuPD-labeled hADMSCs in a non-ischemic mouse (middle column), and AuPD-labeled hADMSCs in a surgical ischemia mouse model (right column). The US images are shown in grayscale map (a.u.) and the PA images are shown in color scale map (a.u.). Scale bar is 5 mm. (b-c) Spectroscopic PA amplitude (700-900 nm) from mice injected intramuscularly with AuPD-labeled hADMSCs in (b) non-ischemic mice (days D0 to D14, n = 6 animals) and in (c) mice with surgically-induced ischemia (D0 to D7, n = 6 animals). (d) Spectroscopic PA amplitude (700 - 900 nm) from mice injected intramuscularly with AuPD-labeled hADMSCs, measured longitudinally (days D0, D7 and D14) in the non-ischemic and the surgical ischemia models. (e) Quantitative PA amplitude values (800 nm) measured in mice receiving intramuscular injections of: Matrigel in non-ischemic animals (days D0 to D7, n=3 animals), and AuPD-labeled hADMSCs in non-ischemic mice and in the surgical ischemia model (days D0 to D14, n=6 animals). Lines shown in spectra are means averaged over all animals within each group while shaded regions represent standard deviations.

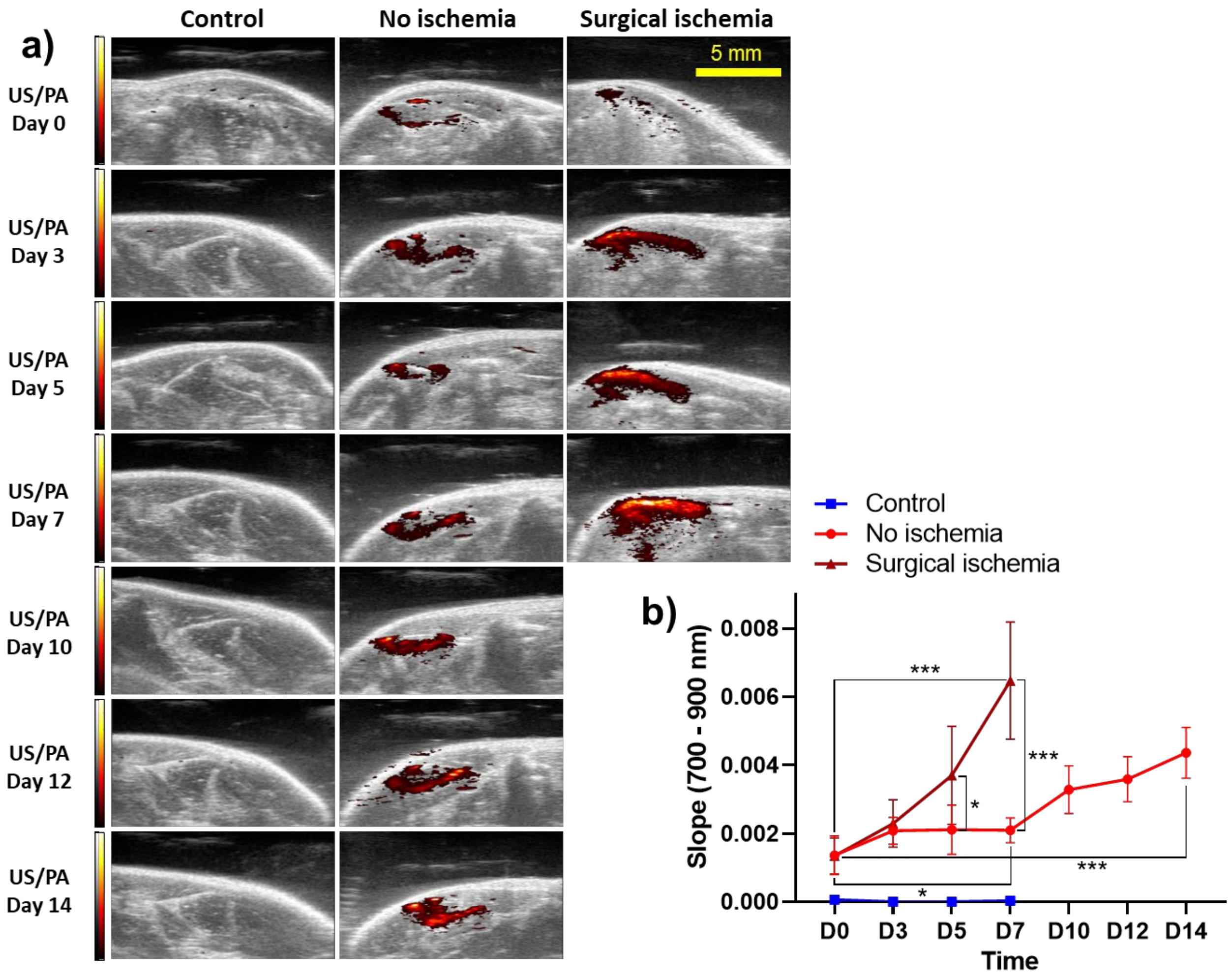

Figure S15. Characterization of the activity of the AuPD nanosensor *in vivo* using spectral slope analysis. (a) Representative images of spectral slopes (see main text) derived from the PA spectra (700-900 nm) from mice injected intramuscularly with: Matrigel into a non-ischemic mouse (left column), AuPD-labeled hADMSCs into a non-ischemic mouse (middle column), and AuPD-labeled hADMSCs into a surgical ischemia model (right column). Scale bar is 5 mm. (b) Spectral slope values derived from PA spectra (700 – 900 nm) as shown in panel (a) vs. time. Data were analyzed by two-factor ANOVA with replication on days and ischemic condition. The days, ischemic condition, and their interaction were significant (p-values 1.9×10^{-8}, 1.0×10^{-6}, 1.8×10^{-6} respectively). This was followed by post hoc testing using Dunnett's test (* < 0.05, ** < 0.01, *** < 0.001)

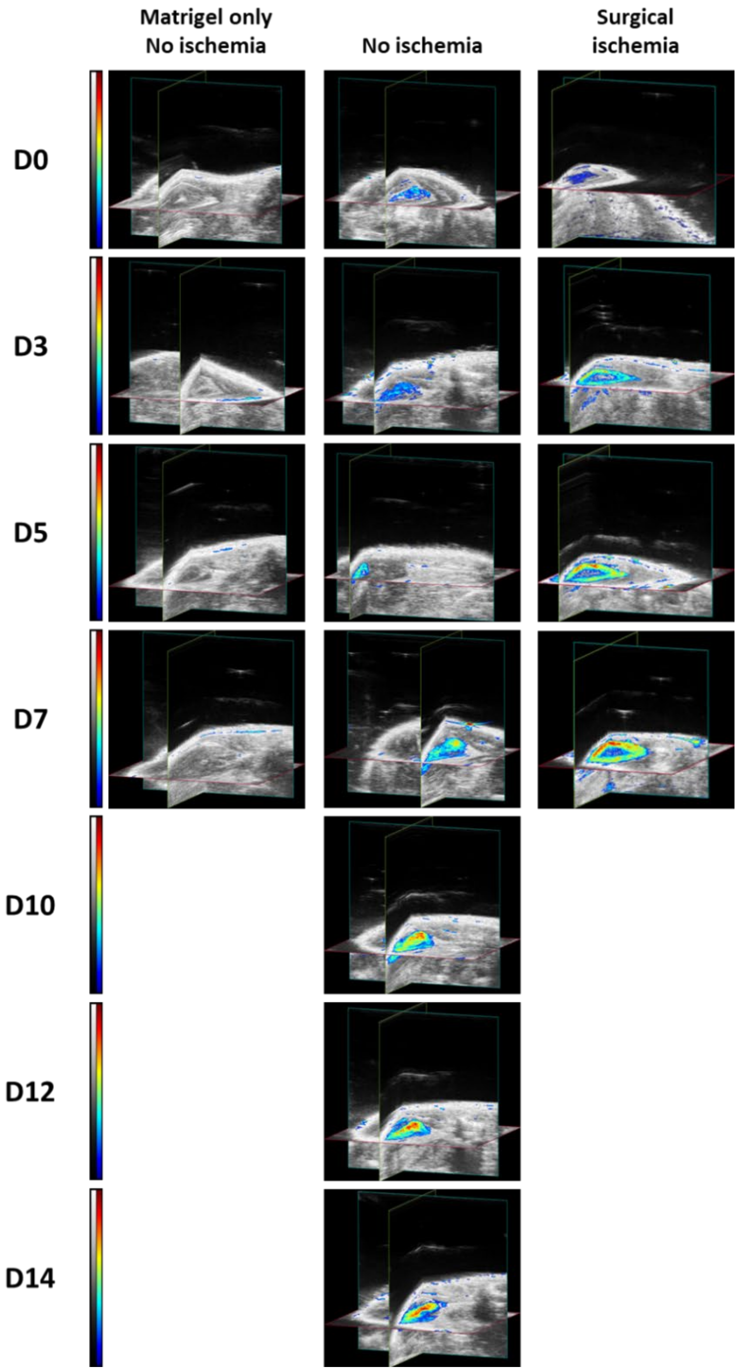

Figure S16. 3D characterization of the activity of the AuPD nanosensor *in vivo*. Cross-sectional views derived from representative 3D PA/US images from mice receiving intramuscular injections of: Matrigel in a non-ischemic mouse (left column), AuPD-labeled hADMSCs in a non-ischemic mouse (middle column), and AuPD-labeled hADMSCs in a surgical ischemia mouse model (right column), from day 0 to day 14. All PA images were captured at 800 nm. The representative slices display the transverse, coronal, and sagittal slices chosen to showcase the region of interest in the injection area, allowing us to visualize the 3D distribution.

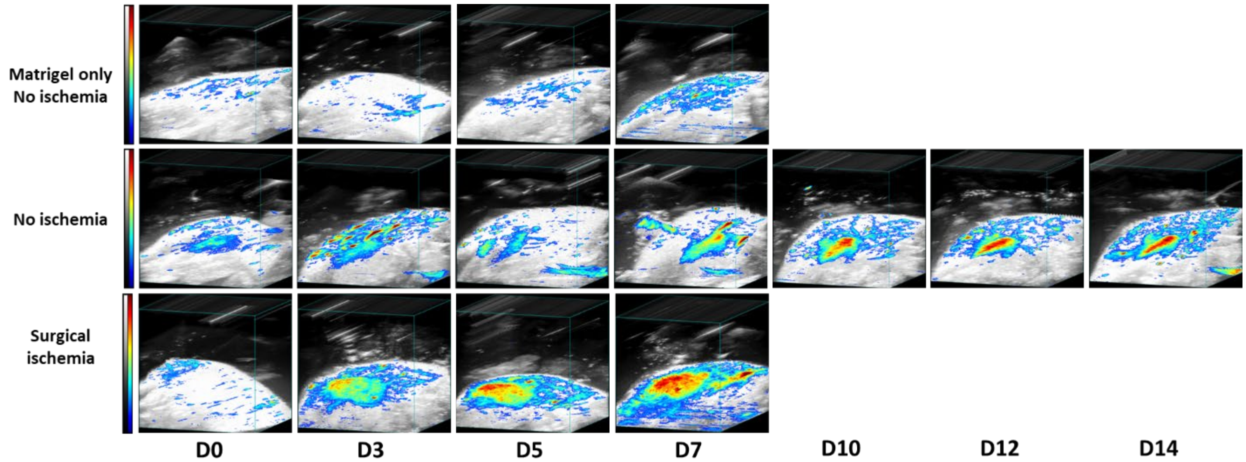

Figure S17. Characterization of the activity of the AuPD nanosensor *in vivo*. Maximum intensity projections derived from representative 3D PA/US images taken from mice receiving intramuscular injections of: Matrigel in in a non-ischemic mouse (top row), AuPD-labeled hADMSCs in a non-ischemic mouse (middle row), and AuPD-labeled hADMSCs in a surgical ischemia mouse model (bottom row) from day 0 to day 14. All PA images were captured at 800 nm. The front face of the 3D image is 13.94 mm in width and 15 mm in depth. The depth of each 3D image "cube" depended on the number of slices acquired, which varied for each experimental case and timepoint due to different stem cell spread after injection. Each slice is 0.154 mm and the number of image slices for each condition were:

- Matrigel only control with no ischemia D0 – 157 slices
- Matrigel only control with no ischemia D3 – 131 slices
- Matrigel only control with no ischemia D5 – 157 slices
- Matrigel only control with no ischemia D7 – 157 slices
- AuPD-labeled hADMSCs with no ischemia D0 – 184 slices
- AuPD-labeled hADMSCs with no ischemia D3 – 184 slices
- AuPD-labeled hADMSCs with no ischemia D5 – 184 slices
- AuPD-labeled hADMSCs with no ischemia D7 – 184 slices
- AuPD-labeled hADMSCs with no ischemia D3 – 184 slices
- AuPD-labeled hADMSCs with no ischemia D5 – 184 slices
- AuPD-labeled hADMSCs with no ischemia D7 – 184 slices
- AuPD-labeled hADMSCs with surgical ischemia D0 – 157 slices
- AuPD-labeled hADMSCs with surgical ischemia D3 – 157 slices
- AuPD-labeled hADMSCs with surgical ischemia D5 – 171 slices
- AuPD-labeled hADMSCs with surgical ischemia D7 – 236 slices

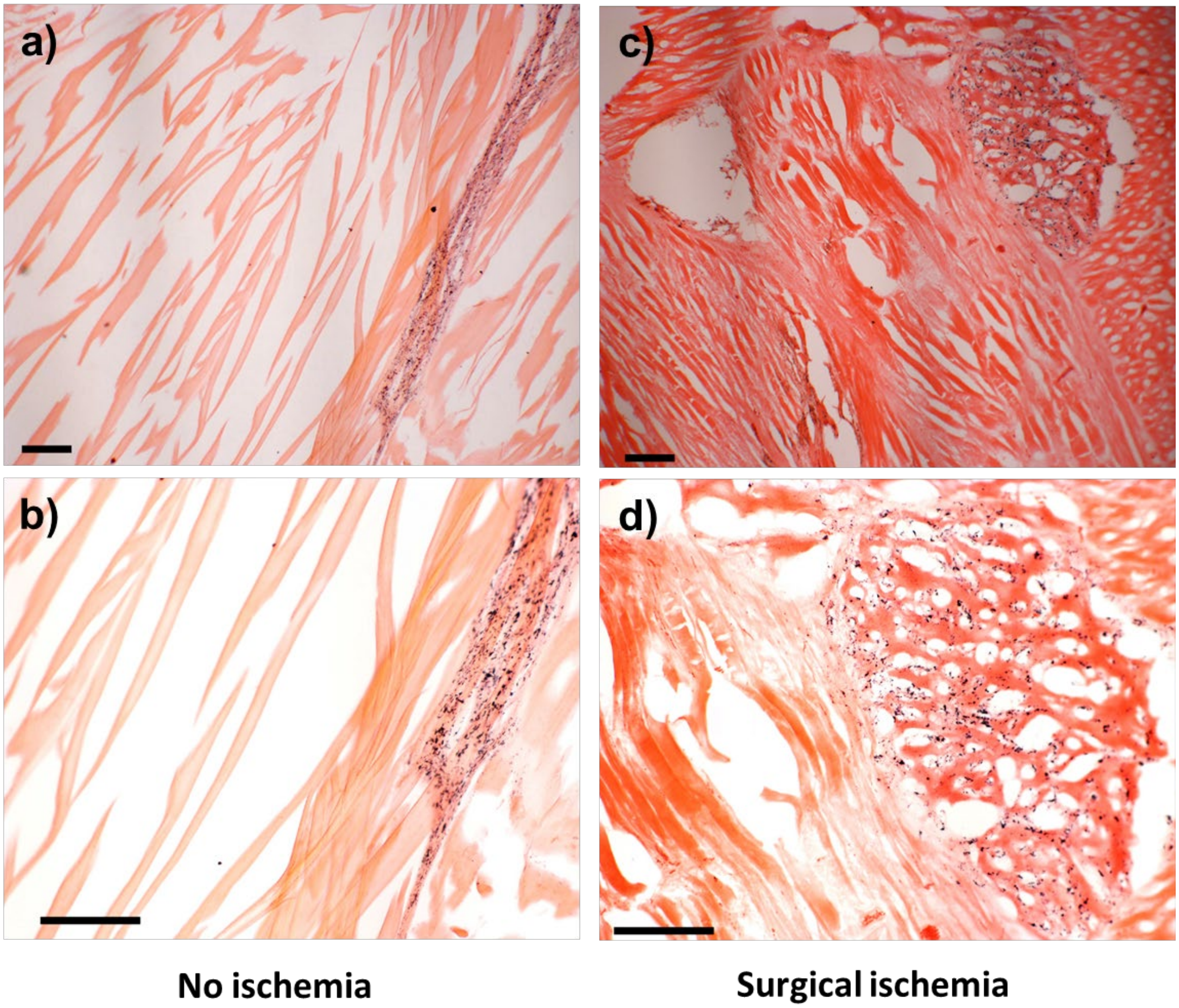

Figure S18. (a, b) Histologic images of hindlimb muscle stained with eosin from a representative non-ischemic mouse injected with AuPD-labeled hADMSCs (scale bars = 200 μm). (c, d) Similar to (a, b), but from a mouse with surgical ischemia. Purple dots indicate AuPD nanosensor aggregates and confirm the presence of the nanosensor in the muscle tissue. We also observed morphological signs of muscle damage in the form of irregular gaps and torn muscle fibers in the surgical ischemia hindlimb tissue samples.

Supplementary Note 2. Captions for video 1 and video 2

Video S1. 3D characterization of the activity of the AuPD nanosensor *in vivo*. Representative serial transverse US/PA images (800 nm) from a 3D data set of mice injected intramuscularly with: Matrigel-only control in a non-ischemic mouse (left column), AuPD-labeled hADMSCs in a non-ischemic mouse (middle column), and AuPD-labeled hADMSCs in a surgical ischemia mouse model (right column). The US images are shown in grayscale map (a.u.) and the PA images are shown in color scale map (a.u.). Each frame is a 0.154 mm slice. Different conditions and days have varying number of slices due to different stem cell spread after injection. The number of slices for each condition are:

- Matrigel only control with no ischemia D0 – 157 slices
- Matrigel only control with no ischemia D3 – 131 slices
- Matrigel only control with no ischemia D5 – 157 slices
- Matrigel only control with no ischemia D7 – 157 slices
- AuPD-labeled hADMSCs with no ischemia D0 – 184 slices
- AuPD-labeled hADMSCs with no ischemia D3 – 184 slices
- AuPD-labeled hADMSCs with no ischemia D5 – 184 slices
- AuPD-labeled hADMSCs with no ischemia D7 – 184 slices
- AuPD-labeled hADMSCs with surgical ischemia D0 – 157 slices
- AuPD-labeled hADMSCs with surgical ischemia D3 – 157 slices
- AuPD-labeled hADMSCs with surgical ischemia D5 – 171 slices
- AuPD-labeled hADMSCs with surgical ischemia D7 – 236 slices

Video S2. Spectral characterization of the activity of the AuPD nanosensor *in vivo*. Representative spectral transverse US/PA images from mice injected intramuscularly with: Matrigel-only control in a non-ischemic mouse (left column), AuPD-labeled hADMSCs in a non-ischemic mouse (middle column), and AuPD-labeled hADMSCs in a surgical ischemia mouse model (right column). PA images were acquired from 680 nm to 970 nm at 5 nm wavelength intervals. The center transverse slice was chosen to show maximal stem cell area.

REFERENCES

(1) Müller-Ehmsen, J.; Whittaker, P.; Kloner, R. A.; Dow, J. S.; Sakoda, T.; Long, T. I.; Laird, P. W.; Kedes, L. Survival and Development of Neonatal Rat Cardiomyocytes Transplanted into Adult Myocardium. *Journal of Molecular and Cellular Cardiology* **2002**, *34* (2), 107–116. https://doi.org/10.1006/jmcc.2001.1491.

(2) Dhada, K. S.; Hernandez, D. S.; Suggs, L. J. In Vivo Photoacoustic Tracking of Mesenchymal Stem Cell Viability. *ACS Nano* **2019**, *13* (7), 7791–7799. https://doi.org/10.1021/acsnano.9b01802.

(3) Sheikh, A. Y.; Huber, B. C.; Narsinh, K. H.; Spin, J. M.; van der Bogt, K.; de Almeida, P. E.; Ransohoff, K. J.; Kraft, D. L.; Fajardo, G.; Ardigo, D.; Ransohoff, J.; Bernstein, D.; Fischbein, M. P.; Robbins, R. C.; Wu, J. C. In Vivo Functional and Transcriptional Profiling of Bone Marrow Stem Cells after Transplantation into Ischemic Myocardium. *Arterioscler Thromb Vasc Biol* **2012**, *32* (1), 92–102. https://doi.org/10.1161/ATVBAHA.111.238618.

(4) Haider, H. K.; Ashraf, M. Strategies to Promote Donor Cell Survival: Combining Preconditioning Approach with Stem Cell Transplantation. *J Mol Cell Cardiol* **2008**, *45* (4), 554–566. https://doi.org/10.1016/j.yjmcc.2008.05.004.
